# Heterogeneous Interfaces of Ni_**3**_Se_**4**_ Nanoclusters Decorated on a Ni_**3**_N Surface Enhance Efficient and Durable Hydrogen Evolution Reactions in Alkaline Electrolyte

**DOI:** 10.1021/jacs.4c17747

**Published:** 2025-05-06

**Authors:** Dessalew Dagnew Alemayehu, Meng-Che Tsai, Meng-Hsuan Tsai, Chueh-Cheng Yang, Chun-Chi Chang, Chia-Yu Chang, Endalkachew Asefa Moges, Keseven Lakshmanan, Yosef Nikodimos, Wei-Nien Su, Chia-Hsin Wang, Bing Joe Hwang

**Affiliations:** † Nano-electrochemistry Laboratory, Graduate Institute of Applied Science and Technology, National Taiwan University of Science and Technology, Taipei 106, Taiwan; ‡ Nano-electrochemistry Laboratory, Department of Chemical Engineering, National Taiwan University of Science and Technology, Taipei 106, Taiwan; § National Synchrotron Radiation Research Center (NSRRC), Hsinchu 300092, Taiwan; ∥ Department of Greenergy, National University of Tainan, Tainan 700301, Taiwan; ⊥ Sustainable Electrochemical Energy Development (SEED) Center, National Taiwan University of Science and Technology, Taipei 106, Taiwan

## Abstract

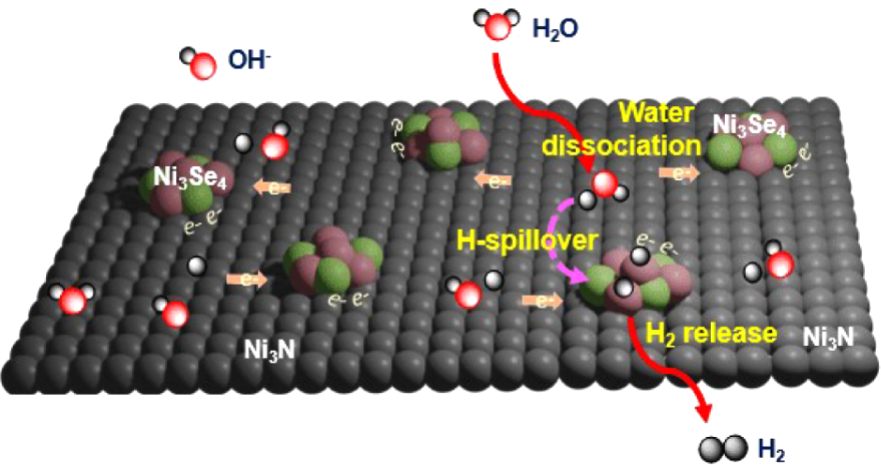

Transition metal selenides (TMSes) have been identified as cost-efficient alternatives to platinum (Pt) for the alkaline hydrogen evolution reaction (HER) owing to their distinct electronic properties and excellent conductivity. However, they encounter challenges such as sluggish water dissociation and severe oxidative degradation, requiring further optimizations. In this study, we developed a dual-site heterogeneous catalyst, Ni_3_Se_4_–Ni_3_N, by decorating Ni_3_Se_4_ nanoclusters on a Ni_3_N substrate. This catalyst design promoted significant interfacial electronic interactions, modulated electronic structures, and enhanced the adsorption of the intermediates. Various spectroscopic analyses and theoretical calculations revealed that the nitride surfaces improved water adsorption and dissociation, enriching the surface with adsorbed hydrogen (H*) atoms, while the Se sites facilitated hydrogen coupling and subsequent release of H_2_. Following a hydrogen spillover mechanism, the surface-adsorbed hydrogen atoms were transferred to nearby electron-dense selenide sites for H_2_ formation and release. Consequently, the optimized catalyst demonstrated improved HER activity, requiring only an ∼60 mV overpotential at 10 mA cm^–2^ current density and maintained stability under higher potential conditions.

## 1 Introduction

The ever-increasing global energy demand is predominantly fulfilled by fossil fuels, which comprise over 80% of the energy mix.^1^ Fossil fuel combustion, however, poses a significant environmental threat due to greenhouse gas emissions.^2,3^ This underscores the urgent need for alternative energy sources, with hydrogen (H_2_) emerging as a prospective carbon-neutral energy technology, offering environmental benefits and high energy density.^3,4^ More than 95% of the world’s hydrogen fuel is generated through coal gasification, partial oxidation, and catalytic steam reforming, notably contributing to increasing global CO_2_ emissions.^5^ Conversely, merely 4% of hydrogen fuel is produced through the environmentally benign and sustainable water-splitting process.^6,7^ Electrocatalysts with a low overpotential are essential for the alkaline hydrogen evolution reaction (HER) to enable scalable production of sustainable hydrogen fuel via water electrolysis. Despite the remarkable catalytic activity of Pt toward HER, its high cost and scarce resource availability significantly limit widespread adoption.^8,9^

Transition metal oxides,^10^ hydroxides,^11^ nitrides,^12^ phosphides,^13^ sulfides,^14^ and selenides^15^ have shown remarkable activity, making them cost-effective substitutes to noble metal-based catalysts. Among these, transition metal selenides are considered as one of the potential catalysts for HER because of their exceptional electronic properties and superior electrical conductivity.^16,17^ The superior HER activity of selenides is also due to the partial negative charge centered on Se atoms, which enhances their capacity to capture protons.^18^ For instance, Xie and coworkers accounted for the increased HER performance of the cubic-CoSe_2_ (c-CoSe_2_) phase to the increased electron localization on Se atoms, which facilitates hydrogen adsorption and conversion into hydrogen molecules as compared to the orthorhombic-CoSe_2_ (o-CoSe_2_) phase.^19^ More importantly, the suitable hydrogen adsorption energy of selenides, revealed by both theoretical and experimental studies, is another potential attribute of their compelling HER activity. Wang and coworkers investigated that Se sites in NiSe_2_ have much lower hydrogen adsorption energy than Ni sites, making these Se sites the primary contributors to the outstanding HER activity of Se-enriched NiSe_2_.^20^ More recent studies also revealed the continuous charge distribution near the Fermi level in both NiSe_2_ and Ni_3_Se_4_, the latter exhibiting relatively higher distribution at the Fermi level.^21^ Nonetheless, transition metal selenides have encountered challenges such as sluggish reaction kinetics, a limited number of active sites, a tendency to agglomerate, and rapid deactivation in strong electrolytes.^22^ This underscores the need for innovative material designs to fully exploit their beneficial properties for widespread use in electrocatalysis. Various notable approaches, such as phase modulation, heterostructure formation, vacancy engineering, and element doping, have been employed to achieve optimal catalytic performances.^23−26^

Heterointerface formulations are considered to be the most promising approach to enhance the inherent catalytic activity of selenides. For instance, a MoSe_2_–Cu_2_S heterointerface exhibited higher HER performance credited to the regulated electronic states and increased number of active sites achieved by decorating MoSe_2_ inert basal planes with Cu_2_S nanocrystals.^27^ Similarly, the Cu-doped Ru/RuSe_2_ heterogeneous electrocatalyst exhibited outstanding catalytic HER performance assigned to optimized interfacial electronic states that enhance intermediate adsorption.^28^ In more recent studies, Banerjee and coworkers revealed the catalytic improvements of selenides by engineering Co_0.85_Se/MoSe_2_ heterostructures. It is indicated that Co_0.85_Se optimized water dissociation while MoSe_2_ enhanced H_2_ release, synergistically enhancing the overall performance.^29^ Beyond heterointerface formulations, recent studies have considered the hydrogen spillover mechanism in catalyst design to achieve more effective HER kinetics. Wang et al. investigated the influence of hydrogen spillover on HER efficiency by designing a Pd-decorated CoP catalyst.^30^ They found that Pd improves water dissociation, increases hydrogen adsorption on the catalyst surface, and aids its transfer to the P site of CoP, resulting in a boosted HER performance. In the same way, heterostructure catalysts such as Ru-WO_3–*x*_, Pt/CoP, and Pt/TiO_2_ follow the hydrogen spillover mechanism and exhibit optimal HER activity.^31−33^ Conclusively, many studies have noted the significant role of the hydrogen spillover mechanism in enhancing the HER activity. Beyond activity, stability is a key factor to consider in designing effective electrocatalysts. Recently, transition metal nitrides (TMNs) have been recognized as potential catalyst supports owing to their excellent electrical conductivity (metallic nature), thermal stability, and corrosion resistance under operational conditions.^34^ Moreover, TMNs are also found to enhance water adsorption and dissociation during alkaline HER, attributed to the electron-rich nitrogen on their surfaces.^35^

Considering the abovementioned points, engineering a heterointerface involving TMNs could exploit the merits of nitrides: their superior stability and water dissociation capabilities. In this study, we designed a heterogeneous catalyst by decorating Ni_3_Se_4_ nanoclusters on a Ni_3_N substrate, aiming to enhance water dissociation and enrich the catalyst surface with H* to achieve efficient HER. The electronic state characterizations using X-ray photoelectron spectroscopy (XPS) and X-ray absorption spectroscopy (XAS) revealed improved interfacial charge transfer and optimized electronic states. Meanwhile, in situ and operando ambient pressure XPS measurements demonstrated substantial water adsorption/dissociation on the nitride surfaces. The density functional theory (DFT) computation also suggests enhanced water adsorption/dissociation on Ni_3_N surfaces with enriched surface H* species. These H* species then move to the nearby electron-rich Se center, combining and releasing H_2_ through the hydrogen spillover mechanism. This process enabled the tailored catalyst to exhibit exceptional alkaline HER performance, requiring only ∼60 mV overpotential to achieve a current density of 10 mA cm^–2^ and sustaining stable operation at higher current densities.

## 2 Experimental Section

### 2.1 Chemicals

Nickel(II) nitrate hexahydrate (Ni(NO_3_)_2_·6H_2_O, 99.5%, Acros Organics, USA), urea (CH_4_N_2_O, 99.5%, Thermo Fisher Scientific, USA), ammonium fluoride (NH_4_F, >98%, Thermo Fisher Scientific, USA), hydrochloric acid (HCl, >37%), selenium powder (Se, >99%, Sigma-Aldrich, USA), sodium borohydride (NaBH_4_, >99%, Thermo Fisher Scientific, USA), nickel foam (NF, thickness 1 mm, Sheng Qiang, China), 20 wt % Pt/C (TEC10E50E, Tanaka Kikinzoku Kogyo K.K., Japan), potassium hydroxide (KOH, >85%, Sigma-Aldrich), Nafion solution (86–87%, Aldrich), acetone (C_3_H_6_O, 99.5%), ethanol (EtOH, 99.5%), and DI water (18.2 mΩ cm) were used without further purification.

### 2.2 Materials Synthesis

In this study, the Ni_3_Se_4_–Ni_3_N/NF hybrid electrocatalyst was synthesized through a meticulous two-step process involving topochemical nitridation and selenization. Ni_3_N/NF was initially obtained by hydrothermally growing Ni(OH)_2_ on nickel foam and annealing it at 400 °C with excess urea for 2 h under an argon gas flow. In the second stage, the Ni_3_N/NF was placed in a 100 mL Teflon-lined stainless steel reactor containing 20 mL of 0.01 M aqueous solution of Ni(NO_3_)_2_·6H_2_O, followed by a 30 mL NaHSe solution. The autoclave contents were heated at 150 °C for 6 h and then naturally cooled to room temperature. The Ni_3_N–Ni_3_Se_4_/NF heterostructure was carefully rinsed with deionized water and dried overnight at 50 °C. The Ni_3_Se_4_/NF electrocatalyst was synthesized following a similar procedure described above in the second stage, except that a clean bare NF was used instead of Ni_3_N/NF.

In this procedure, selenium was activated by reacting 65 mg of Se with 70 mg of NaBH_4_ in 5 mL of Ar-saturated deionized water for 1 h under continuous Ar gas flow. Subsequently, the resulting clear NaHSe solution was diluted to 30 mL with ethanol for selenization.

### 2.3 Materials Characterization

The composition and crystal structure of the as-synthesized catalysts were examined using powder X-ray diffraction (PXRD, Bruker D2 Phaser) with a 2-theta range of 10° to 80°. Scanning electron microscopy (EDX JSM 6500F, JEOL) and energy-dispersive X-ray (EDX) analysis with a beam voltage of 15 kV, and transmission electron microscopy (TEM) were employed to examine micromorphologies and heterointerface structures of the synthesized catalysts.

The surface compositions and chemical states were explored through X-ray photoelectron spectroscopy (XPS) at beamline 24A1 Taiwan Light Source (TLS) end station of the National Synchrotron Radiation Research Center (NSRRC). This specialized beamline can deliver soft X-ray photons with energy ranging from 15 to 1600 eV.^36,37^ The XPS spectra of the as-synthesized catalysts were obtained using 1150 eV excitation energies, with the analyzer’s pass energy set to 40 eV. High-resolution scans were conducted in 0.1 eV steps with a dwell time of 100 ms. Spectra acquisition was performed using SpecsLab Prodigy, while spectrum deconvolution was carried out by XPS peakfit. The Au foil with a binding energy of 84 eV was used to calibrate the spectra. To further reveal the subsurface chemical states and electronic transitions, we also collected soft X-ray absorption spectra (s-XAS) at the TLS 24A1 beamline. The local coordination environment and bulk electronic structures of the catalysts were examined by using hard X-ray absorption spectroscopy (h-XAS) at the TLS 17C1 beamline of NSRRC.

We also conducted in situ near-ambient-pressure X-ray photoelectron spectroscopy (NAP-XPS) at the 24A1 TLS beamline to investigate the water adsorption behavior of the synthesized materials at varying water pressures. To obtain an improved signal intensity under high water pressure, an excitation energy of 750 eV, which offers high photon flux, was used during NAP-XPS analysis.

The analyzer pass energy was set to 40 eV, and high-resolution scans were performed with 0.1 eV increments and a dwell time of 100 ms. The chamber base pressure was reduced to 0.5 mbar during the NAP-XPS experiments. For calibration, the XPS spectra were referenced to the Au 4f signal with a binding energy of 84 eV. SpecsLab Prodigy software was used for spectra acquisition, and the spectrum was deconvoluted using XPS Peakfit. Operando NAP-XPS measurements were also carried out to explore the surface electronic structure changes under cathodic potentials in a 1 M KOH electrolyte solution. Further details of the operando measurements can be found in the literature.^36^

### 2.4 Electrochemical Measurement

All of the electrochemical performances were measured in a three-electrode electrochemical cell equipped with a potentiostat (Metrohm, Autolab PGSTAT302N) at room temperature. The HER performances of the as-synthesized catalysts were evaluated in an aqueous solution of KOH (1 M) using a reference electrode of RHE (reversible hydrogen electrode), a graphite rod as a counter electrode, and the catalysts developed on NF directly used as a working electrode. The linear sweep voltammetry (LSV) curves were obtained at a scan rate of 1 mV s^–1^ with *iR* compensation. Tafel plots were derived by fitting the polarization curves between the overpotential (η) and the logarithm of the current density (log *j*). Cyclic voltammetry (CV) curves were measured in the double-layer region (0.1–0.5 V, without Faradaic processes) at different scan rates to quantify the double-layer capacitance (*C*_dl_). The electrochemical surface area (ECSA) can be estimated from the equation ECSA = *C*_dl_/*C*_s_, where *C*_s_ is the specific capacitance, typically ranging from 0.022 to 0.130 mF cm^–2^ in an alkaline electrolyte. Our calculations used a *C*_s_ value of 0.040 mF cm^–2^ based on previous studies.^38^

## 3 Results and Discussion

### 3.1 Synthesis and Characterization of Ni_3_Se_4_–Ni_3_N Heterostructure Electrocatalysts

**Figure 1:**
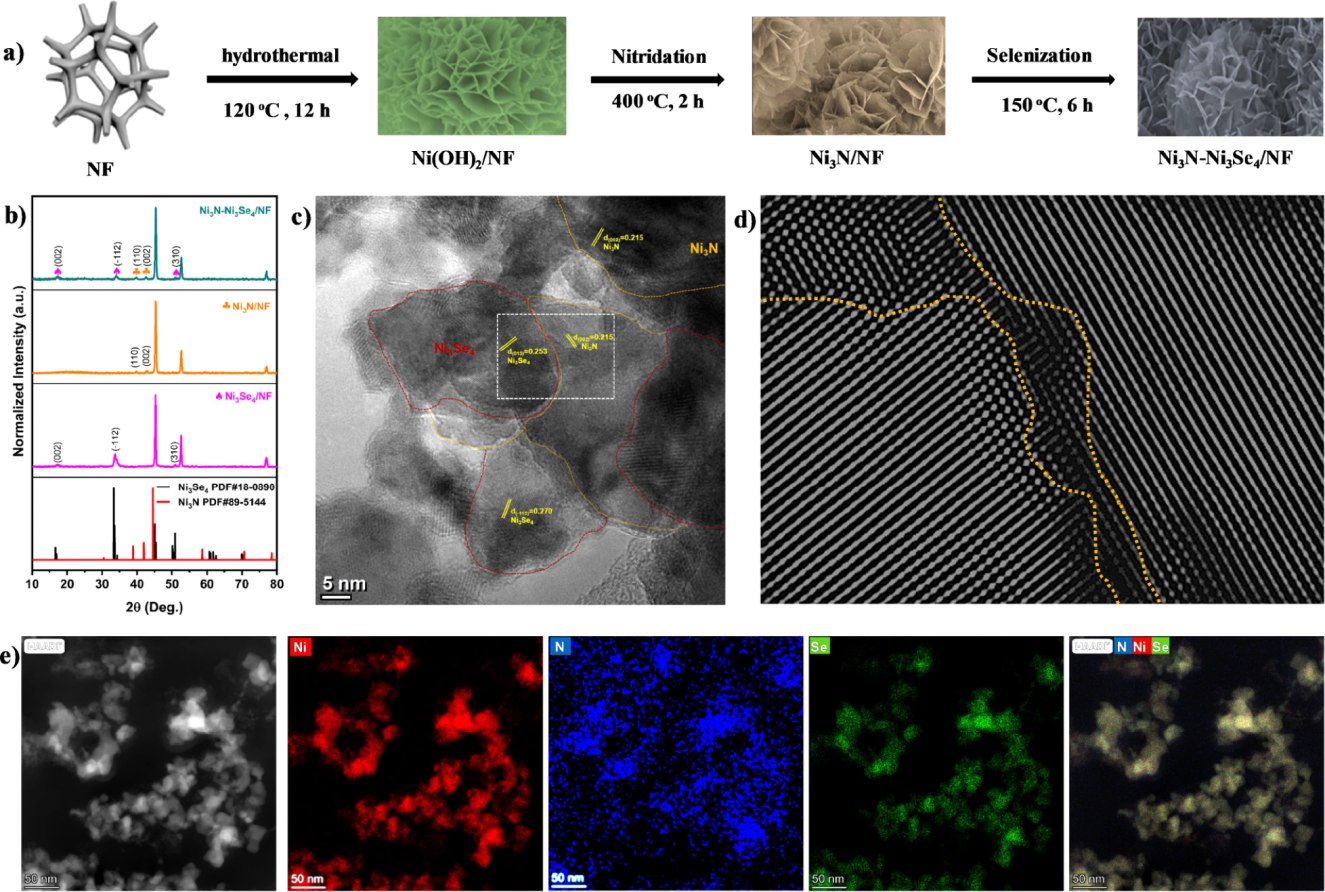
Sample preparation and structural characterization of the Ni_3_Se_4_–Ni_3_N electrocatalyst. (a) Schematic representation of the catalyst synthesis (b) XRD patterns, (c) HR-TEM image, (d) inverse FFT HR-TEM image taken from the selected area in panel c, and (e) STEM-HAADF image and the corresponding EDX elemental mapping images of the Ni_3_Se_4_–Ni_3_N/NF catalyst.

The synthesis pathway of the nickel-foam-supported Ni_3_Se_4_–Ni_3_N electrocatalyst is schematically illustrated in Figure 1a. The dual-site Ni_3_Se_4_–Ni_3_N heterogeneous catalyst was obtained through a two-step process. Initially, Ni_3_N was developed on nickel foam by a topochemical solid–gas reaction in a tube furnace under an Ar/H_2_ atmosphere, where Ni(OH)_2_ grown on the nickel foam reacted with NH_3_ at high temperatures. Subsequently, a Ni_3_Se_4_–Ni_3_N heterogeneous catalyst with abundant interfaces was synthesized through the decoration of Ni_3_Se_4_ nanoclusters on the surface of Ni_3_N/NF via a solvothermal selenization process.

The crystal structure and composition of the Ni_3_Se_4_–Ni_3_N composite electrocatalyst were examined by using XRD measurements. The Ni(OH)_2_/NF precursor exhibits both α and β phases, as illustrated in Figure S1. The α-Ni(OH)_2_ phase is identified by distinct peaks at 11.9°, 23.4°, 34.8°, and 61.4°, while the β-Ni(OH)_2_ phase shows characteristic peaks at 19.9°, 33.8°, 60.2°, and 63.8°. Additionally, the three prominent peaks at 44.5°, 51.8°, and 76.4° are associated with the (111), (200), and (220) crystal planes of the nickel metal substrate (NF). In addition to the peaks associated with metallic nickel, the electrocatalysts grown on nickel foam exhibited distinct diffraction peaks. As depicted in Figure 1b, the primary peaks at 39.74°, 42.83°, and 45.16° correspond to the (110), (002), and (111) lattice planes and can be indexed to hexagonal Ni_3_N (PDF no. 89-5144). Similarly, the weaker yet prominent peaks at 16.6°, 33.3°, 45.36°, and 50.8° are attributed to the (002), (−112), (−114), and (013) planes of monoclinic Ni_3_Se_4_ (PDF no. 18-0890). These XRD findings confirm the successful formation of the Ni_3_Se_4_–Ni_3_N heterostructure.

The microstructures and morphology of the as-synthesized materials were analyzed by utilizing scanning electron microscopy (SEM) and transmission electron microscopy (TEM). The SEM (Figure S2) images revealed the flower-like nanosheet structures of the electrocatalysts. The corresponding energy-dispersive X-ray (EDX) elemental mapping images (Figure S3) demonstrated the homogeneous distribution of Ni, N, and Se elements. The EDX spectrum also confirms the coexistence of these elements (Figure S4). Figure 1c presents a high-resolution TEM (HR-TEM) image showcasing a representative interface between the Ni_3_Se_4_ and Ni_3_N nanostructures. The observed lattice fringe spacings of 2.70 and 2.15 Å correspond to the (−112) planes of Ni_3_Se_4_ and the (002) planes of Ni_3_N, respectively, revealing the formation of an interface between these planes. The inverse fast Fourier transformation (FFT) HR-TEM image (Figure 1d) highlights the boundary region between the Ni_3_Se_4_ and Ni_3_N nanostructures. The high-angle annular dark field (HAADF) image and the corresponding EDX elemental mapping images (Figure 1e) demonstrate Ni, Se, and N distribution surrounding each other.

### 3.2 Electronic Structure and Atomic Coordination

**Figure 2:**
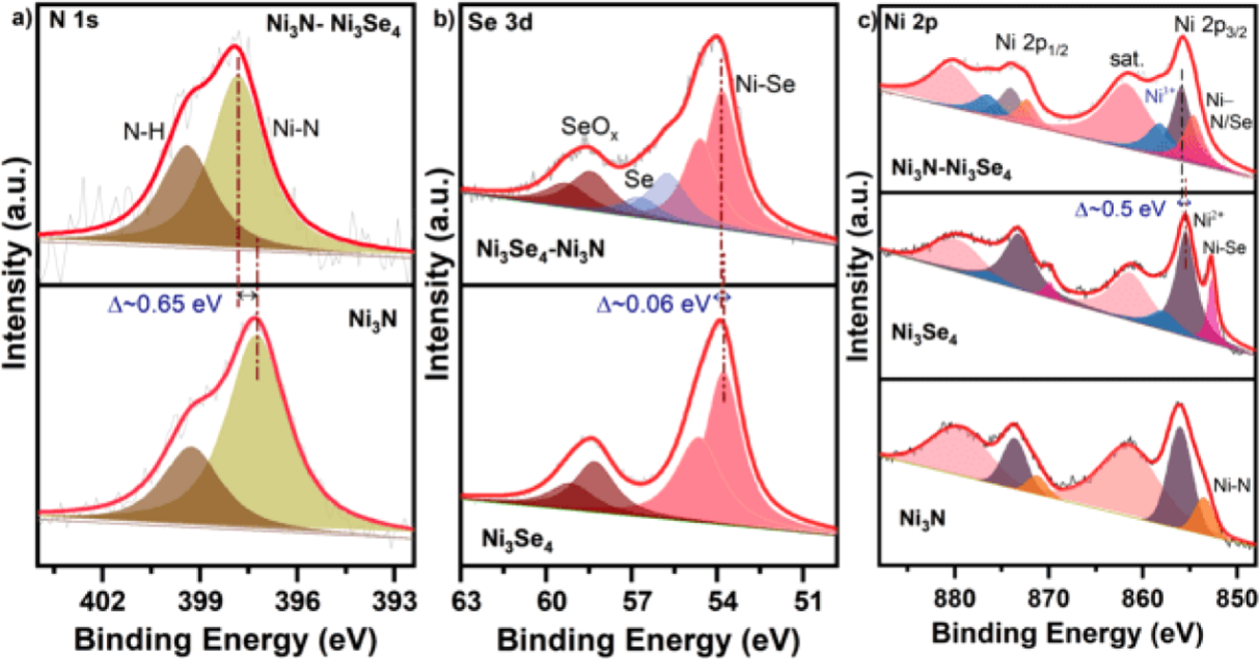
Surface X-ray photoelectron spectroscopy characterization. High-resolution XPS spectra of (a) N 1s, (b) Se 3d, and (c) Ni 2p of the as-synthesized electrocatalysts.

The electronic structure and atomic coordination of the catalysts were thoroughly investigated using high-resolution X-ray photoelectron spectroscopy (HR-XPS), X-ray absorption near-edge structure (XANES) spectroscopy, and extended X-ray absorption fine structure (EXAFS) spectroscopy. The comprehensive survey scan spectra for the synthesized electrocatalysts, as shown in Figure S6, dictated that the catalyst surface consists of Ni, Se, and N elements. The N 1s spectra, shown in Figure 2a, are deconvoluted into two distinct components. A prominent peak at around 397.8 eV corresponds to the Ni–N bond, indicating the formation of nitrides. In comparison, the peak at 399.4 eV is attributed to the N–H bond, resulting from an incomplete reaction with ammonia.^39^ Compared to the N 1s core levels in Ni_3_N, the N 1s peaks in the Ni_3_Se_4_–Ni_3_N heterostructure, particularly the peak assigned to Ni–N bonding, show a positive shift of approximately 0.65 eV. This indicates a higher oxidation state of the surface N anions, suggesting a reduced electron transfer from Ni to N while highlighting strong interfacial charge transfer.^40^Figure 2b depicts the high-resolution Se 3d spectrum of Ni_3_Se_4_ and Ni_3_Se_4_–Ni_3_N samples. The observed peaks at 53.7 and 54.6 eV for Ni_3_Se_4_ can be assigned to Ni–Se binding energies of spin–orbit doublets of Se 3d_5/2_ and Se 3d_3/2_, whereas the peak at ∼58.2 and 59.1 eV corresponds to Se 3d_5/2_ and Se 3d_3/2_ doublets of SeO_*x*_ species, which results from the superficial oxidation of selenides in air.^41^ In the heterostructured Ni_3_Se_4_–Ni_3_N catalyst, the Ni–Se peak appears at 53.8 and 54.7 eV corresponding to spin–orbit doublets. Although the Se 3d spectra exhibit only a slight shift (∼0.06 eV), a new peak associated with Se–Se bonding emerges at ∼55.8 eV in the composite material. Additionally, the intensity of the SeO*_x_* peak decreases upon heterointerface formation. These observations suggest that an interfacial charge transfer induced Se–Se bond formation while reducing the surface oxidized SeO_*x*_ species.

The Ni 2p spectrum presented in Figure 2c features two primary peaks resulting from spin–orbit doublets originating from the 2p_3/2_ and 2p_1/2_ states along with their shake-up satellite peaks. The Ni 2p spectrum of the Ni_3_N sample features Ni–N peaks at ∼853.6 and 871.2 eV, corresponding to the 2p_3/2_ and 2p_1/2_ spin–orbit doublets. Ni^2+^ peaks appear at 856.0 and 873.5 eV due to the superficial oxidation of Ni_3_N. Similarly, the Ni_3_Se_4_ catalyst exhibits Ni–Se peaks at around 852.8 and 870.0 eV, respectively, attributed to the 2p_3/2_ and 2p_1/2_ states. Alongside Ni^2+^ peaks at approximately 855.4 and 873.3 eV, Ni_3_Se_4_ also displays Ni^3+^ peaks at 858.0 and 876.2 eV, corresponding to the same spin–orbit states.

The composite sample’s peaks at ∼854.6 and ∼872.3 eV represent Ni–N/Se bonds for the two spin–orbit doublets. Additional peaks at ∼855.9 and ∼874.0 eV are associated with Ni^2+^ states, while Ni^3+^ peaks are observed around 858.2 and 876.4 eV. Notably, in the Ni 2p spectrum of the Ni_3_Se_4_–Ni_3_N heterointerface catalyst, the Ni^2+^ peak shifts ∼0.5 eV toward higher binding energy compared to the same peak in the Ni_3_Se_4_ spectrum. Overall, the HR-XPS results indicate enhanced oxidation of Ni and N atoms in the heterostructure, highlighting strong interfacial charge transfer while significantly reducing electron flow from Ni to N. The formation of Se–Se bonds, as observed in Se 3d spectra after heterointerface formation, can reduce electron transfer from Ni to N by facilitating interfacial charge transfer. Similar results were investigated where S–S bond formation induced reversed charge flow from the Nb to Ru center in RuS_*x*_/NbS_2_ interfaces.^42^

**Figure 3:**
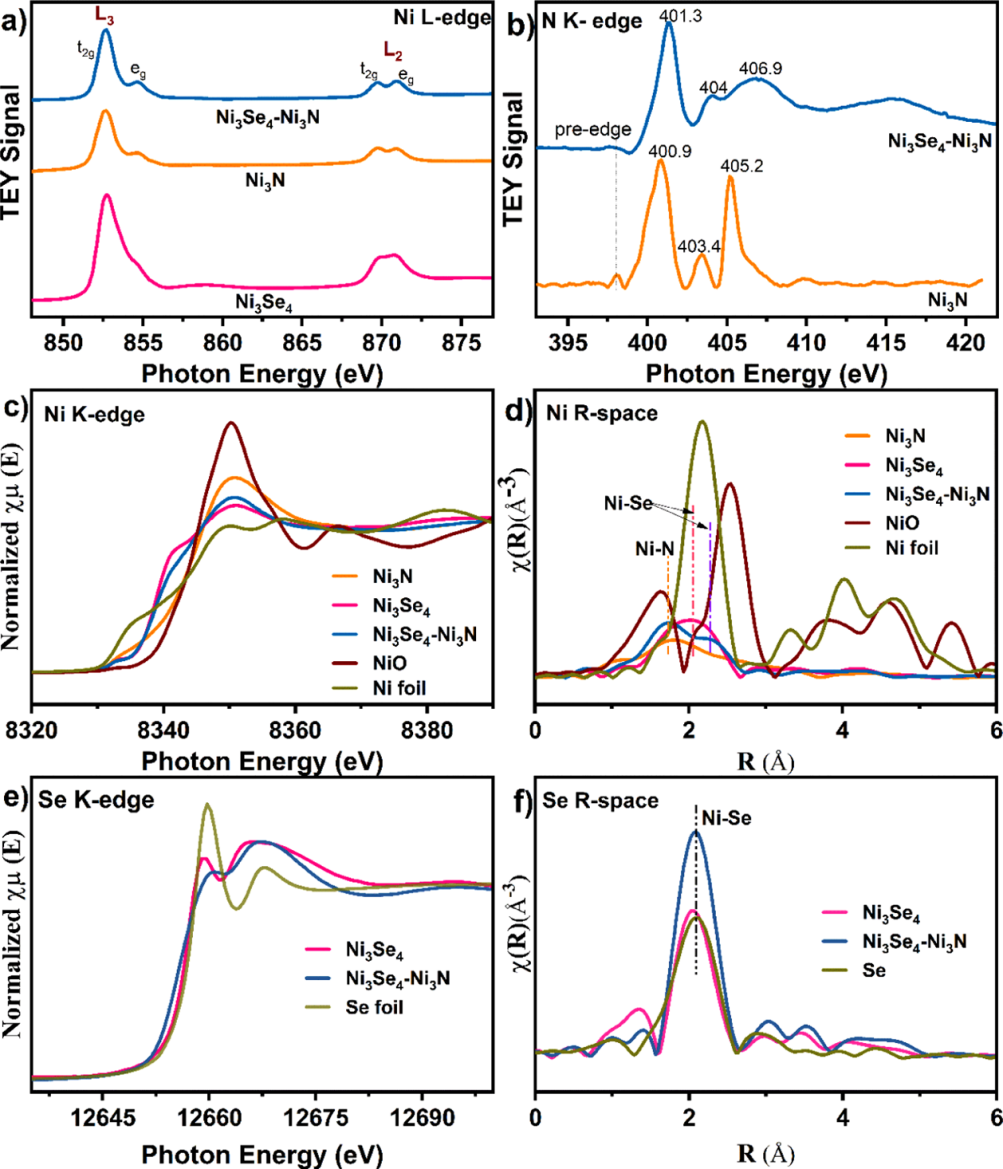
X-ray absorption spectroscopy characterization. (a) Ni L_3,2_-spectra, (b) N K-edge, (c) Ni K-edge, (d) Ni EXAFS, (e) Se K-edge, and (f) Se EXAFS spectra of the as-synthesized electrocatalysts.

To further probe the local electronic structure and reliably determine oxidation states in our materials, we employed soft X-ray absorption spectroscopy (sXAS) operated in total electron yield (TEY) mode (Figure 3a,b). The L-edge XAS is a highly sensitive probe for variations in d-electron occupancy, making it useful for detecting changes in oxidation states and estimating the electron occupancy of t_2g_ and e_g_ orbitals.^43^ As depicted in Figure 3a, the Ni L-edge spectrum features two spin–orbit split regions, L_3_ and L_2_, which correspond to transitions of the Ni 2p_3/2_ and Ni 2p_1/2_ core electrons to 3d orbitals, respectively. The deconvolution of Ni L_3_-edge spectra (Figure S7a) exhibits two distinct peaks with different intensities. The t_2g_ symmetry state is clearly defined at ∼852.6 eV, while the e_g_ symmetry state appears at a slightly higher energy of ∼854.7 eV.^44^ In the Ni_3_Se_4_–Ni_3_N heterostructure catalyst, the fitted peak area ratio of the t_2g_ and e_g_ states is 5.78, noticeably higher than the 4.54 observed for Ni_3_N and the 1.58 observed for Ni_3_Se_4_. These results indicate that the Ni_3_Se_4_–Ni_3_N heterostructure contains a relatively higher concentration of electron holes in the Ni t_2g_ orbitals.

Further evidence of the electronic interactions can be found in the N XANES spectra. As shown in Figure 3b, the N K-edge spectra feature prominent broad peaks within the 399–404.5 eV range, corresponding to electronic transitions to the unoccupied hybridized states of N 2p with Ni 3d t_2g_ and e_g_ orbitals, respectively. Compared with Ni_3_N, the intensity ratio of the t_2g_ and e_g_ peaks in Ni_3_Se_4_–Ni_3_N increased from 6.88 to 13.5 (Figure S7b). The enhanced intensity of the unoccupied t_2g_ spectrum relative to the e_g_ indicates more t_2g_ electron holes in Ni_3_Se_4_–Ni_3_N. Besides the d-orbital electron occupancy, N K-edge spectra display a peak shift toward higher energy after heterointerface formation, which suggests relatively weaker Ni–N interaction and reduced electron transfer from Ni to N.^35^ The soft XAS results revealed the depletion of electrons in the Ni t_2g_ orbital and reduced electron density on nitrogen, pointing out an enhanced interfacial charge transfer to the selenium center. These findings are consistent with XPS results, which confirm enhanced interfacial charge transfer to the selenide component with subsequent localization at Se centers.

The Ni K-edge XANES spectra of Ni_3_Se_4_–Ni_3_N, Ni_3_N, and Ni_3_Se_4_, presented in Figure 3c, reveal significant insights. The pre-edge feature of Ni_3_N shows a slight shift to higher energy, indicating the oxidation of Ni to a higher valence state in Ni_3_N. Conversely, both the Ni_3_Se_4_ and the dual-site catalyst, Ni_3_Se_4_–Ni_3_N, exhibit an absorption edge very close to that of Ni foil, suggesting the electron-dense nature of Ni in selenide and electronic state regulations at heterointerfaces of the Ni_3_Se_4_–Ni_3_N electrocatalyst. Compared to Ni_3_N, the Ni K-edge shifts to lower energy after heterointerface formation, indicating reduced electron transfer from Ni to N. This decrease in Ni-to-N electron transfer is further confirmed by N 1s XPS and N K-edge XAS results, which reveal a lower electron density of nitrogen in the heterostructured catalyst. Moreover, the formation of Se–Se bonds, as observed in the Se 3d XPS, significantly reduces the charge transfer from Ni to N, with excess charge partially localized at the Se center. As a result, Ni in Ni_3_Se_4_–Ni_3_N becomes more electron-rich than Ni in Ni_3_N. The corresponding Fourier transform EXAFS (FT-EXAFS) spectrum for Ni_3_Se_4_–Ni_3_N shows prominent peaks at ∼1.76 Å, which is attributed to the Ni–N scattering feature, and ∼2.30, which accounts for Ni–Se scattering (Figure 3d). The Se K-edge XANES spectrum shown in Figure 3e indicates a partial reduction in the valence state of Se in Ni_3_Se_4_–Ni_3_N compared to Ni_3_Se_4_, as evidenced by the diminished intensity of the white line. This suggests that the observed interfacial electron transfer is partially localized at the Se centers. The Se K-edge FT-EXAFS spectra in Figure 3f also demonstrate a peak at ∼2.04 Å, corresponding to the Ni–Se bond in Ni_3_Se_4_. In the Ni_3_Se_4_–Ni_3_N heterostructure catalyst, this bond is slightly extended to ∼2.08 Å, which is attributed to the electronic structure optimization at the heterointerfaces. These findings align well with the above-described results.

### 3.3 Electrochemical HER Performances

**Figure 4:**
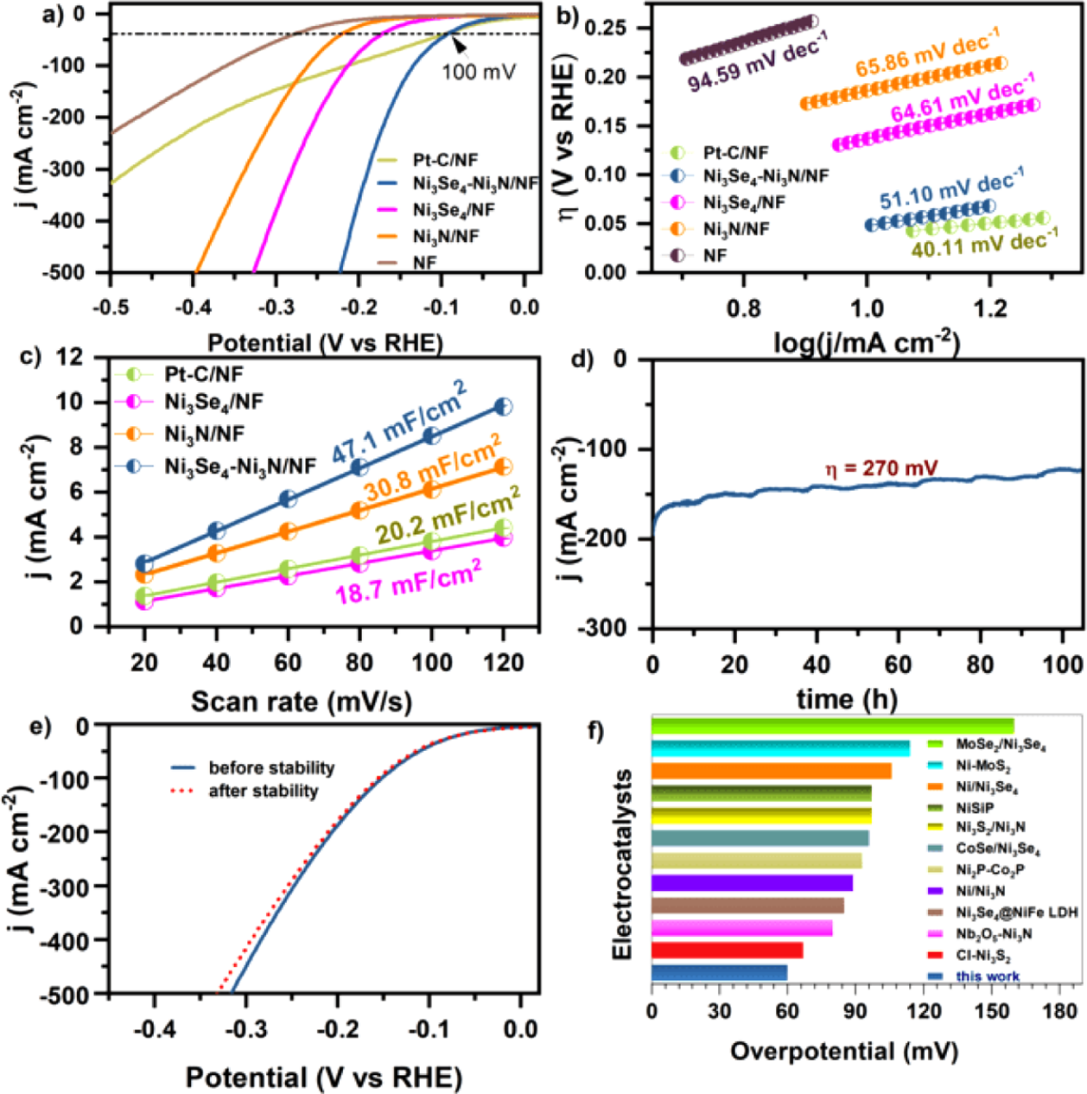
Comparison of the electrochemical performance. (a) *iR*-compensated LSV curves of the electrocatalysts, (b) corresponding Tafel slopes, (c) calculated electrochemical double-layer capacitances, (d) chronoamperometry curve of Ni_3_Se_4_–Ni_3_N at a fixed overpotential of 270 mV, (e) polarization curves before and after chronoamperometry test, and (f) histogram of overpotentials at 10 mA cm^–2^ current density for various electrocatalysts.

The HER activity of the dual-site Ni_3_Se_4_–Ni_3_N heterogeneous catalyst was investigated in an Ar-saturated 1 M KOH electrolyte using a three-electrode setup. For comparison, Ni_3_Se_4_, Ni_3_N, and 20 wt % Pt–C were also tested under identical conditions. As depicted in Figure 4a, Ni_3_Se_4_–Ni_3_N demonstrated a relatively low overpotential of 60 mV to derive a 10 mA cm^–2^ current density, which is significantly lower compared with Ni_3_Se_4_ (η_10_ = 110 mV) and Ni_3_N (η_10_ = 130 mV). Moreover, the designed catalyst demonstrates HER activity comparable to the commercial 20 wt % Pt–C (η_10_ = 42 mV), outperforming the latter at an overpotential exceeding 100 mV. Even though overpotential (η_10_) is the most considered parameter in evaluating the catalytic efficiency of an electrocatalyst, it cannot offer a precise and comprehensive assessment of an electrocatalyst’s efficiency.^45^ Therefore, considering key activity metrics such as turnover frequency (TOF) is essential to scrutinizing the activity of electrocatalysts. The dual-site catalyst demonstrates higher TOF, requiring an overpotential of 145 mV to reach 1 s^–1^, which is significantly lower than that of Ni_3_Se_4_ (257 mV) and Ni_3_N (334 mV), highlighting its outstanding intrinsic catalytic activity (Figure S10b). Additionally, the amount of catalyst loaded greatly impacts activity, so an accurate comparison of catalyst performance requires mass activity—the current normalized by the catalyst mass. It is noted that the tailored heterostructure catalyst has shown relatively higher mass activity comparable with 20 wt % Pt–C (Figure S10a).

To evaluate the microkinetics of the catalysts, the Tafel slopes were determined from the *iR*-compensated LSV polarization curves (Figure 4b). The Ni_3_Se_4_–Ni_3_N exhibited the lowest Tafel slope of 51.1 mV dec^–1^, relative to Ni_3_N (65.86 mV dec^–1^) and Ni_3_Se_4_ (64.61 mV dec^–1^), suggesting the improved HER kinetics of the tailored heterogeneous catalyst (Figure 4b). Electrochemical impedance spectroscopy (EIS) was also used to evaluate the HER kinetics for each sample. The Ni_3_Se_4_–Ni_3_N dual-site electrocatalyst demonstrated a relatively minimal charge transfer resistance (Rct) than Ni_3_Se_4_ and Ni_3_N catalysts (Figure S7 and Table S1), suggesting the enhanced charge transfer rate of Ni_3_Se_4_–Ni_3_N and consequently improved HER activity

To further comprehend the excellent HER performance, we estimated the electrochemical surface area (ECSA) of each catalyst based on its correlation with the double-layer capacitance (*C*_dl_) (Figure S9).^46^ The Ni_3_Se_4_–Ni_3_N catalyst exhibits a higher *C*_dl_ value compared to Ni_3_N and Ni_3_Se_4_, suggesting a higher number of active sites (Figure 4c). It should be noted that Ni_3_Se_4_ outperforms Ni_3_N despite having a lower Cdl or ECSA, which could be due to its high intrinsic activity.^47^ To better reflect the intrinsic activities of the catalysts and minimize geometric effects, the current responses were normalized by ECSA (Figure S10c). After normalization, Ni_3_Se_4_ shows a lower overpotential than Ni_3_N, suggesting its improved intrinsic activity. The long-term performance of the Ni_3_Se_4_–Ni_3_N electrode was evaluated using chronoamperometry at a fixed cell voltage of 270 mV, showing only a minimal current drift after 100 h, highlighting its impressive durability (Figure 4d,e). Additionally, the Ni_3_Se_4_–Ni_3_N catalyst outperformed most of the previously reported TMSe-based electrocatalysts in alkaline HER performance (Figure 4f and Table S2).

### 3.4 Mechanism of HER Enhancement

In order to rationalize the superior HER catalytic performance of the Ni_3_Se_4_–Ni_3_N dual-site electrocatalyst, we primarilyinvestigated water adsorption behavior using NAP-XPS under different water pressures. It should be noted that the kinetics of an alkaline HER is largely limited by the initial Volmer step of water dissociation.^48^ Therefore, an efficient catalyst should optimize water adsorption, allowing the subsequent dissociation process to proceed smoothly. Tailoring the surface electronic structures of electrocatalysts through heteroatom doping, surface defect engineering, heterostructure design, etc. has been revealed to significantly enhance interfacial water adsorption.^49,50^ Using NAP-XPS, we explored how the optimized surface electronic structures of the designed catalyst influenced the initial water adsorption and pinpointed the specific components that facilitate this process. The high-resolution core-level XPS spectra provided details about intricate interactions occurring at the catalyst–water interfaces.

**Figure 5:**
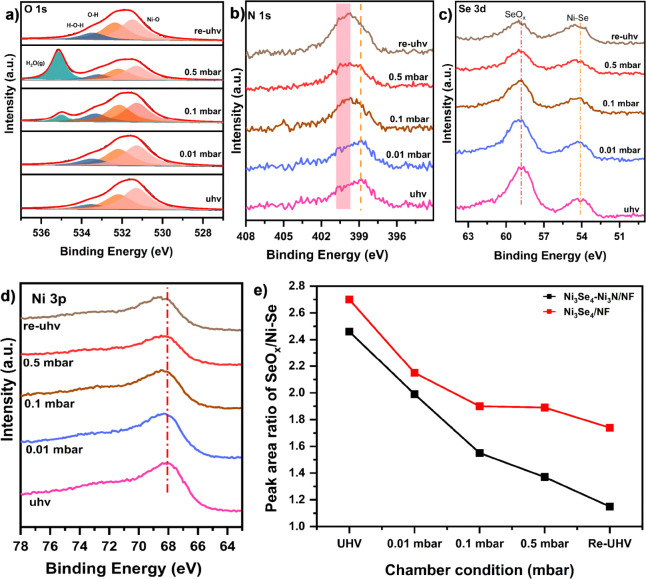
Water adsorption characterizations using near ambient pressure X-ray photoelectron spectroscopy: the HR-XPS spectra of (a) O 1s, (b) N 1s, (c) Se 3d, and (d) Ni 3p of the Ni_3_Se_4_–Ni_3_N catalyst at different water pressures and (e) the peak area ratios of SeO_*x*_/Ni–Se, determined from Se 3d spectra at various surface water coverages.

The O 1s spectra of Ni_3_Se_4_–Ni_3_N dual-site catalyst under ultrahigh vacuum (UHV) and varied water pressures are shown in Figure 5a. The spectra exhibit the components associated with oxide (∼530 eV), hydroxide (∼531.1 eV), and chemisorbed water (∼532.9 eV).^51^ The peak around ∼534 eV, which emerges at a water pressure of 0.1 mbar and becomes more pronounced with increased water coverage, corresponds to water vapor, H_2_O(g). It is noted that the chemisorbed water increases as the level of water in the chamber increases, suggesting enhanced water adsorption on the surfaces of the catalyst. The relative O 1s diagram clearly illustrates the increase in chemisorbed water and the percentage compositions of other components at different surface water coverages (Figure S16). The O 1s spectra for the single-component catalysts are displayed in Figure S15. During NAP-XPS water adsorption measurements, the acquired spectra exhibited a slight positive peak shift, which could arise from the band-bending effect, mostly observed for semiconductor materials at surface/water interactions. It is identified that the adsorption of water molecules induces electron transfer into the catalyst surfaces and bends the energy band downward, causing the peak shift at ambient water pressure.^52^

Similarly, the N 1s spectra hold significant insights into the existence of strong water adsorption on the surface of the catalyst (Figure 5b). The spectrum features a significant increase in the intensity of the peak, mainly associated with the N–H component with increasing surface water coverage. The deconvolution of the spectra demonstrates the expanding peak area for the N–H component as the water pressure on the surface increases (Figure S11). This suggests that the electron-rich nitrogen atoms on the Ni_3_N component facilitate the surface water adsorption process. The calculated percentage of the relative N 1s diagram in Figure S12 shows that the N–H peak area increases by up to 53% at a water pressure of 0.5 mbar. The Ni 3p spectra also show a slightly broadening peak corresponding to the hydroxide layer when exposed to increased water pressure, as depicted in Figure 5d. Thus, it can be concluded that water adsorption on the Ni_3_N surface is promoted through hydrogen bonds formed between the surface nitrogen atoms and the electron-deficient hydrogen atom of the H_2_O molecule. This interaction causes the O of H_2_O molecule to bend significantly toward the surface, facilitating the breaking of the HO–H bond on the Ni atom. The hydrogen atom is easily transferred to the nitrogen atom along the HO–H···N bond on the N–Ni surface, enriching the catalyst with adsorbed hydrogen atoms. Comparable changes in peak intensity were also noted for the Ni_3_N single-component catalyst, while the Ni_3_Se_4_ sample exhibited minimal changes under varying water pressure. The deconvoluted Ni 3p spectra of the samples can be found in Figure S13.

The Se 3d spectra, on the other hand, revealed subtle changes in the surface water levels (Figure 5c). It is evident that with increasing water pressure, the intensity of the peaks corresponding to surface-oxidized selenium (SeO_*x*_) decreases, while the Ni–Se signal is enhanced. This indicates partial reduction of the surface-oxidized selenides with the increasing surface water coverage. This observation suggests the interfacial electron transfer from adsorbed water to the catalyst surface, partially localized at the Se center. A similar anomalous pattern of partial electron transfer from interfacial water to the metal surface is reasoned for the reduced electron density of oxygen and hydrogen atoms at the Pt(111)–water interface.^53^ The deconvoluted spectra presented in Figure S14 clearly depict these changes in the Se species at different surface water levels.

The NAP-XPS analysis revealed an increase in surface water adsorption, characterized by strong HO–H···N bonding and interfacial electron transfer with partial redistribution at the Se center. These robust catalyst–water interactions weaken the O–H bonds and are further destabilized by the interfacial electron transfer, indicating a likelihood of dissociation of the surface-adsorbed water. It is noteworthy that in solid–water interfaces, water adsorption can be either dissociative or nondissociative (molecular water adsorption) depending on the surface electronic structure and intrinsic properties of materials. For instance, surface defects and surface excess electrons play a crucial role in the dissociation of water on semiconducting binary oxides, such as TiO_2_ surfaces.^54,55^ In our study, multiple spectroscopic analyses confirmed substantial interfacial charge transfer. This optimized surface electronic structure enhances the polarization of adsorbed water, inducing dissociative water adsorption on the designed catalyst under high surface water coverage. Moreover, surface structural changes of materials exposed to water are also used to probe dissociative adsorption of water. Wang and coworkers, for instance, attributed the enhanced V^4+^ signal observed in AP-XPS to the dissociation of water adsorbed at the BiVO_4_ surface.^56^ Interestingly, our tailored heterogeneous catalyst exhibited notable surface structural changes with increasing levels of water exposure. The NAP-XPS characterization exhibited structural modifications of surface selenides with increasing surface water coverage, which can be induced by the dissociation of a portion of surface-adsorbed water. To clearly depict surface structural changes, the peak areas of Ni–Se and SeO*_x_* under different chamber conditions were thoroughly analyzed. Notably, the SeO*_x_*/Ni–Se area ratio changes significantly with higher water levels, suggesting structural modifications of surface-exposed species (Figure 5e). The substantial decrease of the SeO_*x*_ peak intensity, along with the strengthened Ni–Se peak upon water adsorption, suggests electron transfer to the catalyst surface, inducing the dissociation of surface-adsorbed water. It should be pointed out that an amorphous surface oxide layer can induce similar effects, though less pronounced, in the single-component Ni_3_Se_4_ catalyst. It is evident that the surface structural changes upon water exposure appear to stabilize at 0.1 mbar water vapor pressure, suggesting limited surface modification compared to dual-site heterogeneous interface structures.

**Figure 6:**
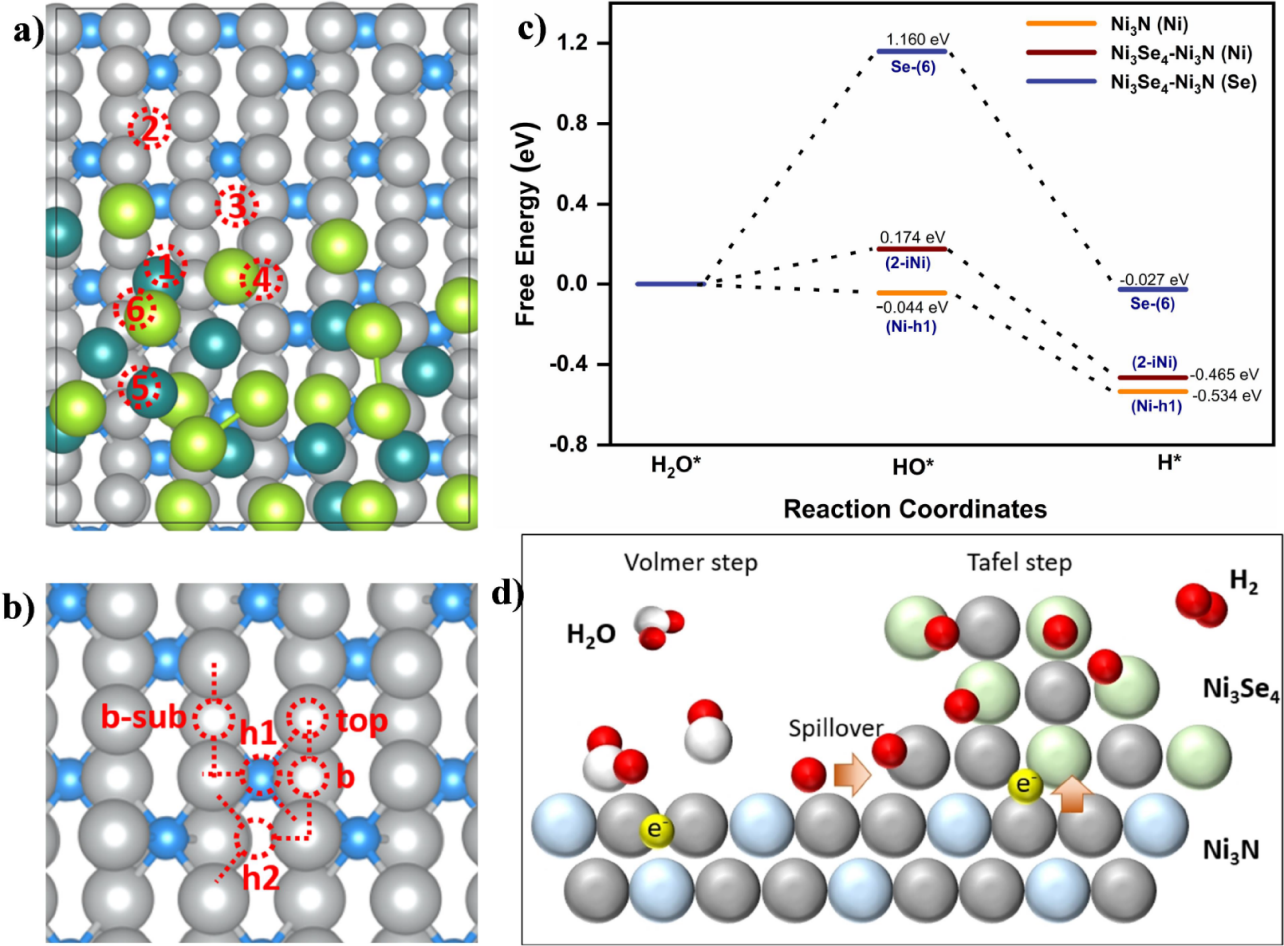
Density functional theory calculations: atomic sites and optimized models of (a) Ni_3_Se_4_–Ni_3_N, (b) Ni_3_N used for DFT calculations, (c) adsorption Gibbs free energy diagram, and (d) the schematic diagram for the proposed HER mechanism. The blue and light green spheres correspond to the N and Se atoms. Both gray and dark green spheres represent Ni in Ni_3_N and Ni_3_Se_4_ for clarity in the top view in the DFT atomic models.

The density functional theory (DFT) calculations were carried out to explore interactions of intermediates further and validate the promoted water dissociation over the Ni_3_N surface. We employed Ni_3_N (110), which forms heterointerfaces with Ni_3_Se_4_ nanoclusters, as a theoretical model for DFT computations to determine the hydroxyl (Δ*G*_OH*_) and hydrogen (Δ*G*_H*_) adsorption-free energies in alkaline solutions. As depicted in Figure 6, the adsorption-free energies of intermediates were determined for different Ni sites in the optimized atomic models of Ni_3_Se_4_–Ni_3_N (110) (Figure 6a) and Ni_3_N (110) (Figure 6b), and the results are given in Table S3. The lowest hydroxyl (Δ*G*_OH*_) and hydrogen (Δ*G*_H*_) adsorption-free energies are found to be −0.044 and −0.534 eV at the Ni-hallow site (Ni-h1) of Ni_3_N (110) surface, respectively. After decorating the surface of a Ni_3_N thin film with Ni_3_Se_4_ nanoclusters to form Ni_3_Se_4_–Ni_3_N heterointerfaces, the minimum adsorption-free energies were determined to be 0.174 eV for hydroxyl (Δ*G*_OH*_) and −0.465 eV for hydrogen (Δ*G*_H*_) at interfacial Ni sites on the Ni_3_N surface. The H* adsorption on nitrogen in both the Ni_3_N (110) and Ni_3_Se_4_–Ni_3_N (110) surfaces was calculated to be −0.705 eV and −0.707 eV, respectively (Figure S17a,b and Table S3). These values indicate strong H* adsorption, which is beneficial for promoting water dissociation. According to the Brønsted–Evans–Polanyi (BEP) relationship, this strong adsorption of H/OH suggests a low water dissociation barrier.^57^ In contrast, the Se sites in the heterostructure exhibited favorable hydrogen adsorption energy (Δ*G*_H*_ = −0.027 eV), suggesting appropriate hydrogen evolution sites. The free energy diagram in Figure 6c illustrates Se as an appropriate site for H_2_ formation and release, while Ni suitably binds the OH* intermediate. The results strongly support our water adsorption NAP-XPS findings, which identify significant water adsorption/dissociation at the Ni_3_N surface. At the same time, H_2_ formation and release are favored at the Se site in the designed catalyst.

Building on the results described, we proposed a hydrogen spillover mechanism to explain the enhanced HER activity observed in alkaline electrolytes (Figure 6d). Our water adsorption studies revealed a strong interaction between water molecules and the catalyst surface, facilitating their dissociation and significantly increasing the amount of H* atoms, particularly at nitride surfaces. Additionally, multiple spectroscopic analyses demonstrated interfacial charge transfer and electronic redistribution at the selenium centers. Consequently, the designed catalyst, featuring a nitride component with higher H* surface coverage and an electron-rich selenide, potentially follows a hydrogen spillover HER mechanism, where adsorbed hydrogen migrates from the nitride surface to selenide sites to form and release H_2_.^58^ Thus, our proposed mechanism involves three key steps: H_2_O adsorption/dissociation, hydrogen spillover, and the formation and release of H_2_.

To further evidence the hydrogen spillover phenomenon’s occurrence, we conducted a H/D kinetic isotope effects (KIEs) experiment in 1 M KOH/D_2_O solution.^59,60^ The LSV curves of the electrocatalysts were acquired in 1 M KOH/H_2_O and 1 M KOH/D_2_O solutions, and the KIE was determined as the ratio of current densities (). The results indicated that hydrogen transfer plays a role in the rate-limiting steps for all of the electrocatalysts. The LSV curves of the electrocatalysts acquired in 1.0 M KOH aqueous electrolyte and 1.0 M KOH D_2_O electrolyte are displayed in Figure S18. The corresponding KIE values were calculated at the selected potentials that drive 50, 100, 150, and 200 mA cm^–2^ current densities in 1 M KOH/H_2_O electrolyte (Table S4). The KIE values for all electrocatalysts are >1, confirming that proton transfer is involved in the rate-limiting steps.^59,61^ Compared with Ni_3_N/NF and Ni_3_Se_4_/NF, the Ni_3_Se_4_–Ni_3_N/NF heterointerface catalyst exhibited lower KIE values, indicating improved hydrogen transfer kinetics. This enhancement is likely due to strong interfacial charge transfer and electron redistribution to the Se center, which promoted the hydrogen spillover process in the dual-site heterostructured electrocatalyst.^42^

**Figure 7:**
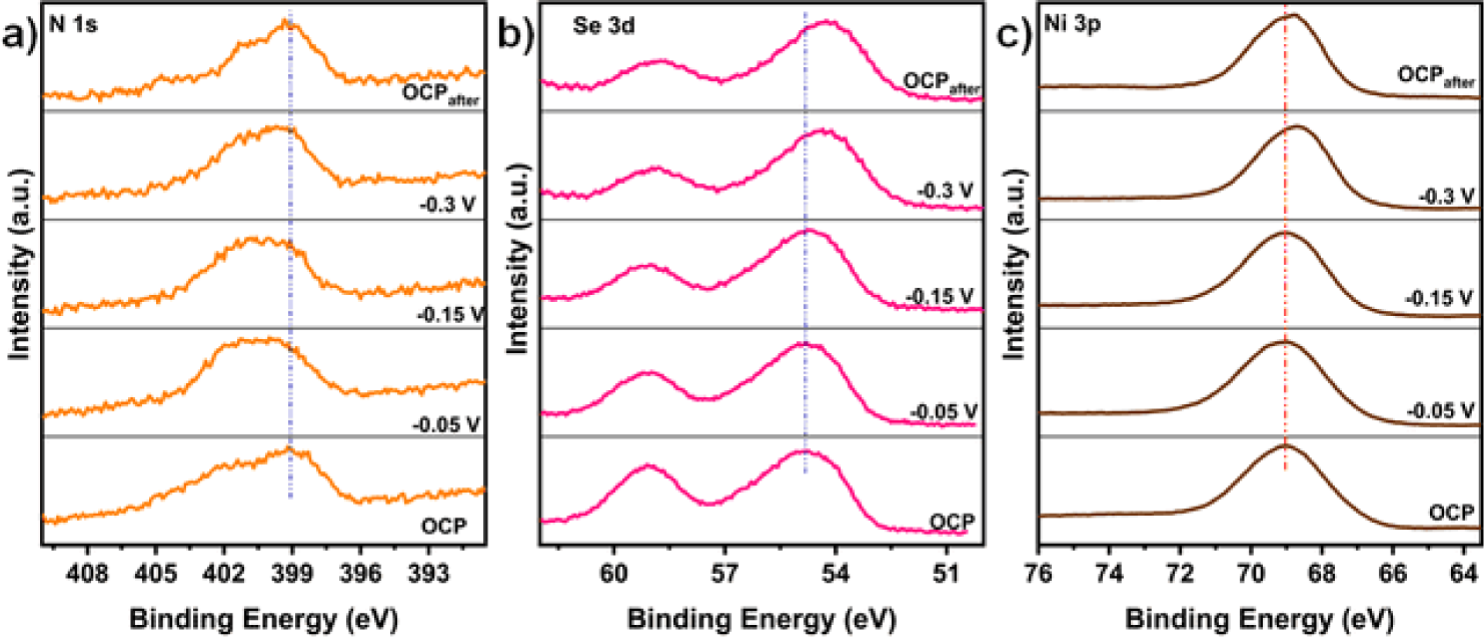
In situ XPS characterization. HR-XPS spectra for (a) N 1s, (b) Se 3d, and (c) Ni 3p acquired under different applied cell voltages in 1 M KOH aqueous solution.

Moreover, we performed operando XPS measurements employing a polymer-based electrochemical setup in an APXPS chamber to gain further insights into the reaction mechanism. The detailed experimental design for operando electrochemical studies has been reported in refs (36) and (37). The operando XPS measurements allowed us to monitor the dynamics of the surface electronic structure and changes in valence states during the reaction. These findings align closely with the in situ NAP-XPS water adsorption results. As illustrated in Figure 7, the peaks at open-circuit conditions appear at slightly higher binding energies, likely due to band-bending effects. This phenomenon is particularly notable when interactions at the electrolyte/electrode interface alter the surface energy of the materials.^45^ Nonetheless, the acquired spectra demonstrate the dynamic evolution of the surface electronic structure with varying cell voltages. The N 1s spectra in Figure 7a show a notable increase in the peak intensity associated with N–H bonding as the cathodic cell voltage increases. The spectra show a slight positive shift upon applying a cathodic potential. This shift is noted as stable at increasing negative potentials without undetectable changes, corroborating the stable surface states after the first evolution during the HER testing.

During operando NAP-XPS characterization, the Ni 3p spectra exhibited relatively broad peaks that remained relatively stable when a voltage of −0.3 V was applied (Figure 7c). A slight negative shift noted after −0.3 V applied voltage suggests the reduction of Ni, which may be partly caused by the reduction of the surface oxide layer under a negative potential. In addition, under strongly negative HER cell voltages, the formation of electron-rich Se can induce the formation of surface Ni–Se species and cause the Ni 3p peak to shift to a relatively lower energy. Nonetheless, despite a slight negative shift under an electron-rich environment, no peaks for new species are observed, indicating that Ni has not undergone significant structural changes. In contrast, the Se 3d spectra exhibit a slight negative shift with increasing cathodic potential, while the SeO_*x*_ peak intensity decreases at more negative potentials (Figure 7b). This points to electron redistribution on Se, partially reducing the amount of Se, which could be a favorable site for hydrogen coupling and H_2_ release. These findings, similar to the in situ water adsorption results, indicate that water adsorption and dissociation primarily occur at the Ni_3_N component of the customized binary catalyst. The hydrogen spillover effect promotes the HER by transferring H* from the nitride surfaces, rich in adsorbed hydrogen, to electron-dense Se sites, where recombination and subsequent H_2_ release are favored.

## 4 Conclusion

We designed a multifunctional heterogeneous catalyst to enhance alkaline HER by embedding Ni_3_Se_4_ nanoclusters on a Ni_3_N substrate. Multiple spectroscopic investigations revealed the specific roles of each component in the dual-site catalyst for improved performance. This catalyst design effectively integrates active sites for water dissociation (Ni_3_N site) and hydrogen recombination (Ni_3_Se_4_ site) with robust electronic structure modulation at the interfaces, resulting in an efficient alkaline HER. This catalyst configuration promotes substantial interfacial charge transfer and fine-tunes the electronic states, resulting in optimized intermediate adsorption. In situ NAP (near ambient pressure)-XPS and operando XPS results confirm that nitride significantly enhances water adsorption and dissociation, while the selenide component supports H_2_ formation and release. The tailored catalyst operates through a hydrogen spillover mechanism where hydrogen atoms adsorbed on nitride surfaces transfer to selenide sites for H_2_ generation. Consequently, the catalyst demonstrated compelling HER requiring as low overpotential as ∼60 mV to derive a 10 mA cm^–2^ current density and operating stably at higher potential conditions.

## Supporting Information Available

Supporting SEM, TEM, EDX results, XPS, and electrochemical measurements and experimental procedures and details (PDF)
