# Supplementary material for: Heterogeneous Interfaces of Ni3Se4 Nanoclusters Decorated on a Ni3N Surface Enhance Efficient and Durable Hydrogen Evolution Reactions in Alkaline Electrolyte
Source: J Am Chem Soc. 2025 May 6;147(19):16047–59. doi: 10.1021/jacs.4c17747 (PMC12082632; doi:10.1021/jacs.4c17747)
Supplement: Supplementary file 1 — ja4c17747_si_001.pdf [file ja4c17747_si_001.pdf]

SUPPORTING INFORMATION

Heterogeneous Interfaces of Ni_3Se_4 Nanoclusters Decorated on Ni_3N Surface Enhance Efficient and Durable Hydrogen Evolution Reactions in Alkaline Electrolyte

Dessalew Dagnew Alemayehu[1,3], Meng-Che Tsai[2*], Meng-Hsuan Tsai[3], Chueh-Cheng Yang[3], Chun-Chi Chang[1,3], Chia-Yu Chang[1,3], Endalkachew Asefa Moges[2], Keseven Lakshmanan[2], Yosef Nikodimos[2], Wei-Nien Su[1,2,*], Chia-Hsin Wang[3,*], Bing Joe Hwang[1,2,3*]

[1]Nano-electrochemistry Laboratory, Graduate Institute of Applied Science and Technology, National Taiwan University of Science and Technology, Taipei 106, Taiwan

[2]Nano-electrochemistry Laboratory, Department of Chemical Engineering, National Taiwan University of Science and Technology, Taipei 106, Taiwan

[3]National Synchrotron Radiation Research Center (NSRRC), Hsinchu 300092, Taiwan

Experimental Section

Chemicals

Nickel(II) nitrate hexahydrate ($Ni(NO_3)_2 \cdot 6H_2O$, 99.5% Acros Organics, USA), urea (CH_4N_2O, 99.5%, Thermoscientific, USA), ammonium fluoride (NH_4F, >98%, Theromoscientific, USA), Hydrochloric acid (HCl, >37%), Selenium powder (Se, >99%, sigma aldrich, USA), Sodium borohydride ($NaBH_4$, >99%, Theromoscientific, USA), Nickel foam (NF, thickness 1 mm, Sheng Qiang, China), 20 wt% Pt/C (TEC10E50E, Tanaka Kikinzoku Kogyo K. K., Japan), potassium hydroxide (KOH, >85%, Sigma-Aldrich), Nafion solution (86–87%) (Aldrich), Acetone (C_3H_6O, 99.5%,), ethanol (EtOH, 99.5%) and DI water (18.2 mΩ cm) were used without further purifications.

Preparation of 20 wt% Pt-C/NF

5.0 mg of commercial 20 wt% Pt/C catalysts was dissolved in 1200 μL of ethanol and 800 μL of water. Then 30 μL of 5 wt% Nafion solution was added into the mix and ultrasonicated for 30 minutes to form a homogenous ink. This uniform catalyst ink was then drop casted onto the cleaned NF and dried in air. Pt-C/NF loading was estimated to be 0.25 mg cm^{-2}. The mass loadings of all the synthesized catalysts were determined as the weight difference between the bare substrate (NF) and the substrate after catalyst growth to account for mass effects on their performance.

Estimation of Electrochemical Active Surface Area.

Cyclic voltammetry (CV) measurements were conducted at various scan rates in the non-faradaic regions to determine the electrochemical double-layer capacitance (Cdl). These Cdl values were

then used to estimate the electrochemical surface area (ECSA). To calculate Cdl, the differences between the positive and negative current densities (j = (ja - jc)/2) at a specific potential were plotted against the scan rates of the CV. The slope of this linear plot represents the Cdl. By applying the specific capacitance value for a flat standard with a surface area of 1 cm^2, Cdl can be converted into the electrochemically active surface area (ECSA). The specific capacitance of a flat surface typically ranges between 20 and 60 F cm^{-2}.[1-3] We use a 40 μFcm^{-2} assumption to calculate ECSA. Equation (S1) can be used to compute the ECSA:

$$A_{ECSA} = \frac{C_{dl}\, of\ catalyst\ (mF\ cm^{-2})}{0.04\ mF\ cm^{-2}\ per\ cm^2_{ECSA}} \quad \text{(S1)}$$

Computational Details

All the calculations were performed with the Vienna Ab initio Simulation Package (VASP) code 5.4.4 based on the density-functional theory (DFT).[4] The generalized gradient approximation (GGA) with the Perdew-Burke-Ernzerhof (PBE) functional was used for the differential charge density, and the ion electron interaction was described by the projector augmented wave (PAW) method with a cutoff energy of 500 eV.[5] The structural models of Ni_3N (110), and Ni_3Se_4-Ni_3N (110) surfaces were adopted to calculate the adsorption free energy of the reaction intermediates in hydrogen evolution reaction (HER). Furthermore, the DFT-D_3 correction method in Grimme's scheme was used to describe the long-range vdW interactions accurately. All the calculations were carried out until the total energy and force were less than 10^{-5} eV per atom and 0.05 eV $Å^{-1}$, respectively.

The calculation of the free energy for the reaction intermediates was performed by an equation:

$$\Delta G = \Delta E + \Delta ZPE - T\Delta S$$

where E is the total energy, ZPE is the zero-point energy, T is the temperature in Kelvin, and S is the entropy. Calculating the vibrational frequencies in the harmonic normal-mode approximation determines the value of ZPE.

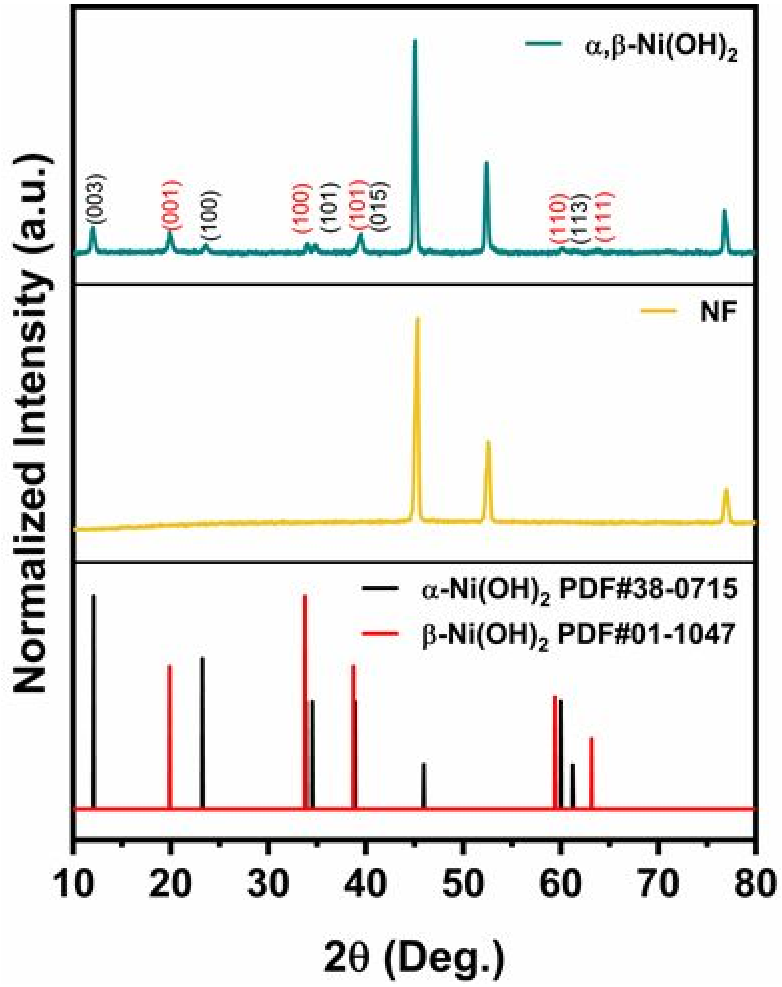

Figure S1. X-ray diffraction (XRD) patterns for the $Ni(OH)_2$ precursor and Nickel Foam (NF) substrate

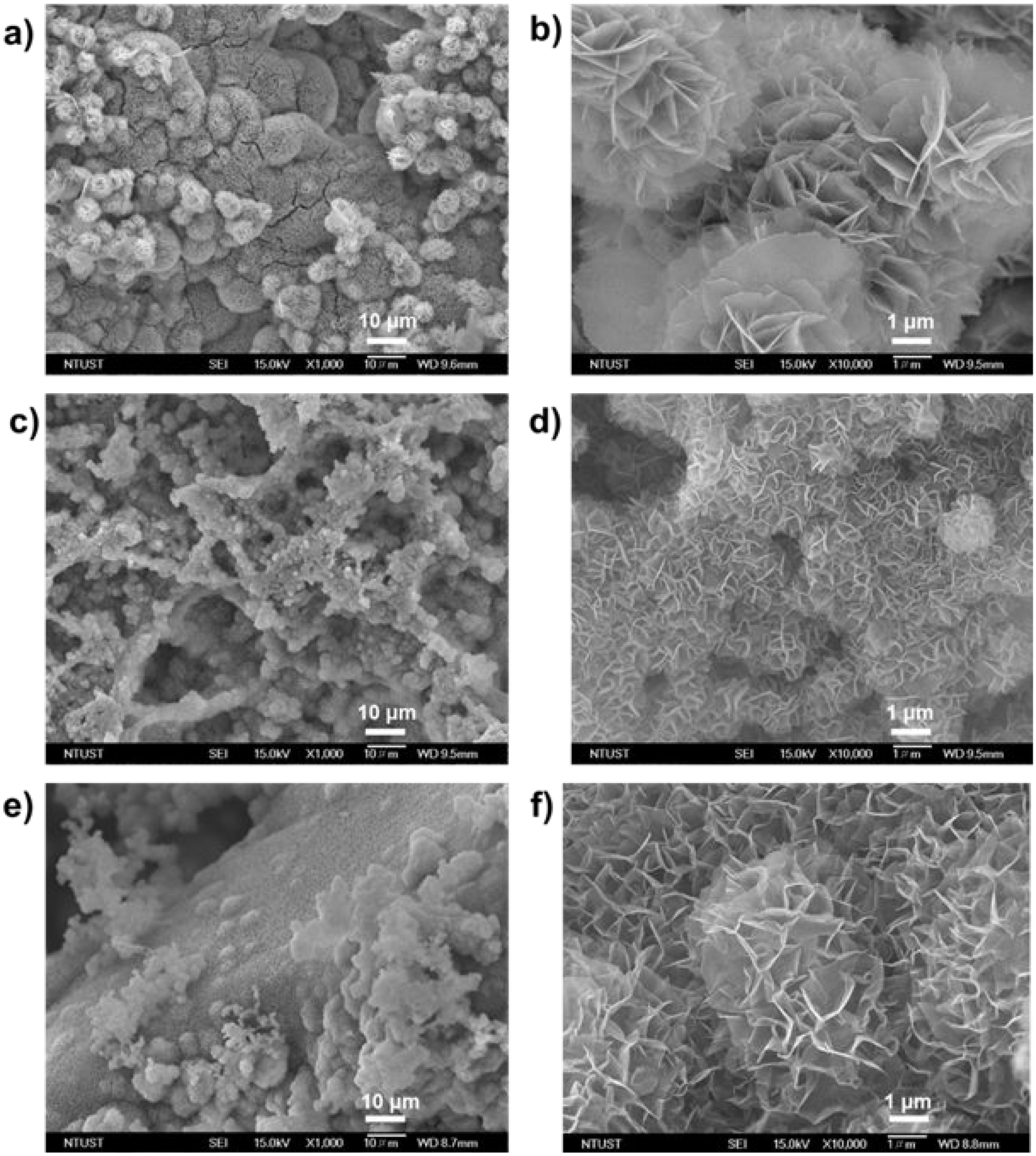

Figure S2. SEM images of (a, b) Ni_3N/NF, (c, d) Ni_3Se_4/NF and (e, f) Ni_3Se_4-Ni_3N/NF catalysts at different resolutions.

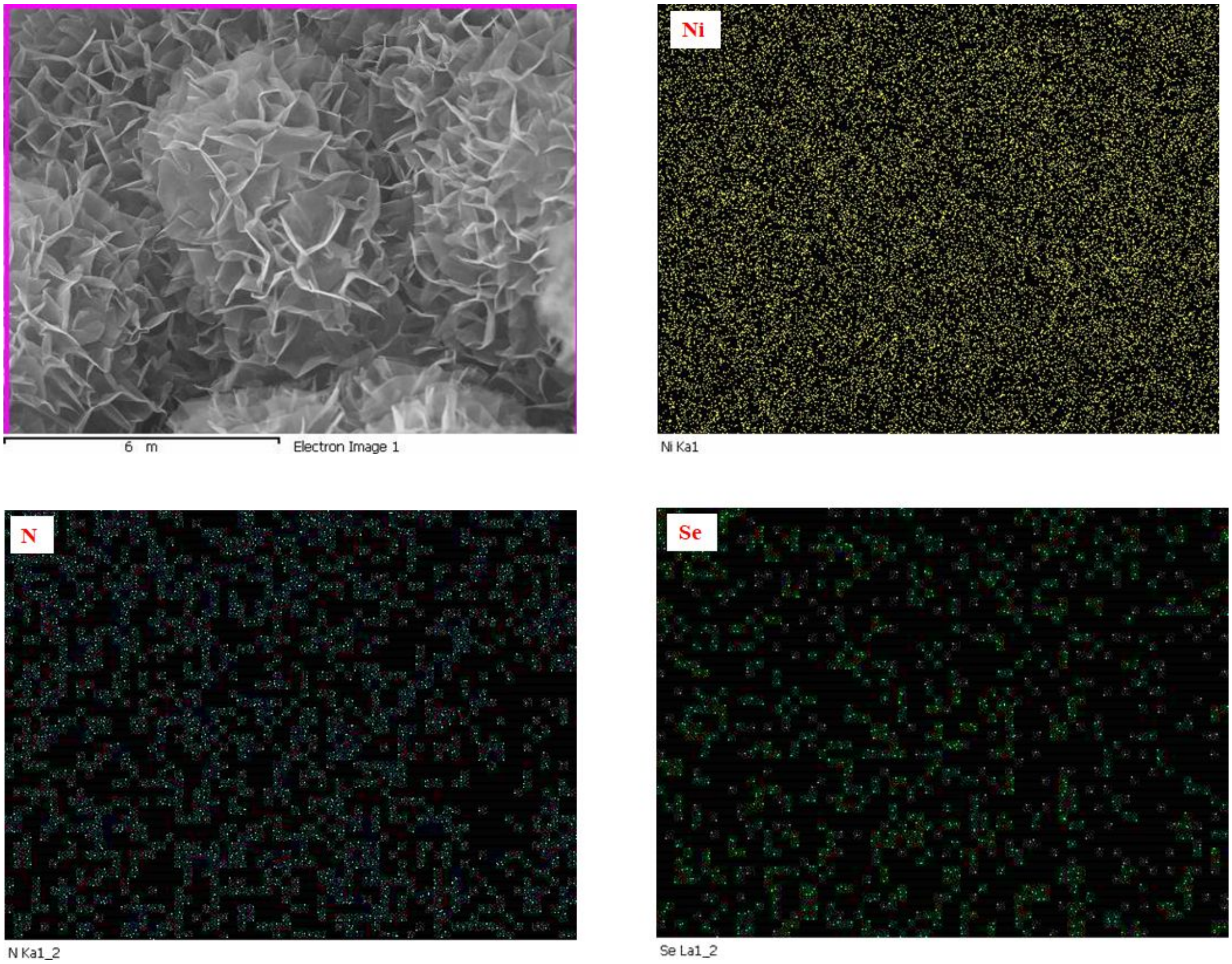

Figure S3. SEM image and the corresponding EDS elemental mapping for Ni_3Se_4-Ni_3N/NF catalyst

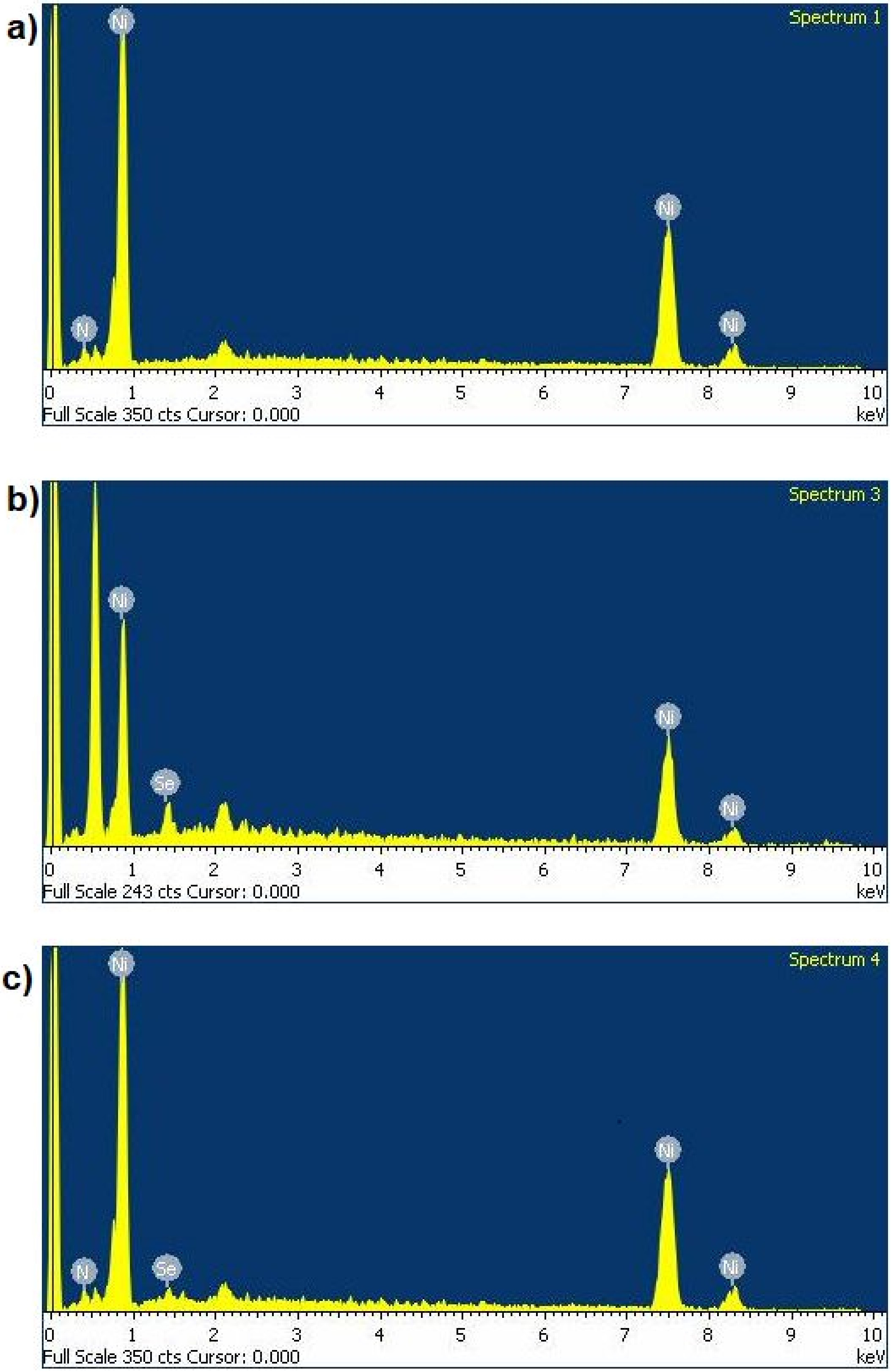

Figure S4. EDX spectra of (a) Ni_3N/NF, (b) Ni_3Se_4/NF and (c) Ni_3Se_4-Ni_3N /NF catalysts

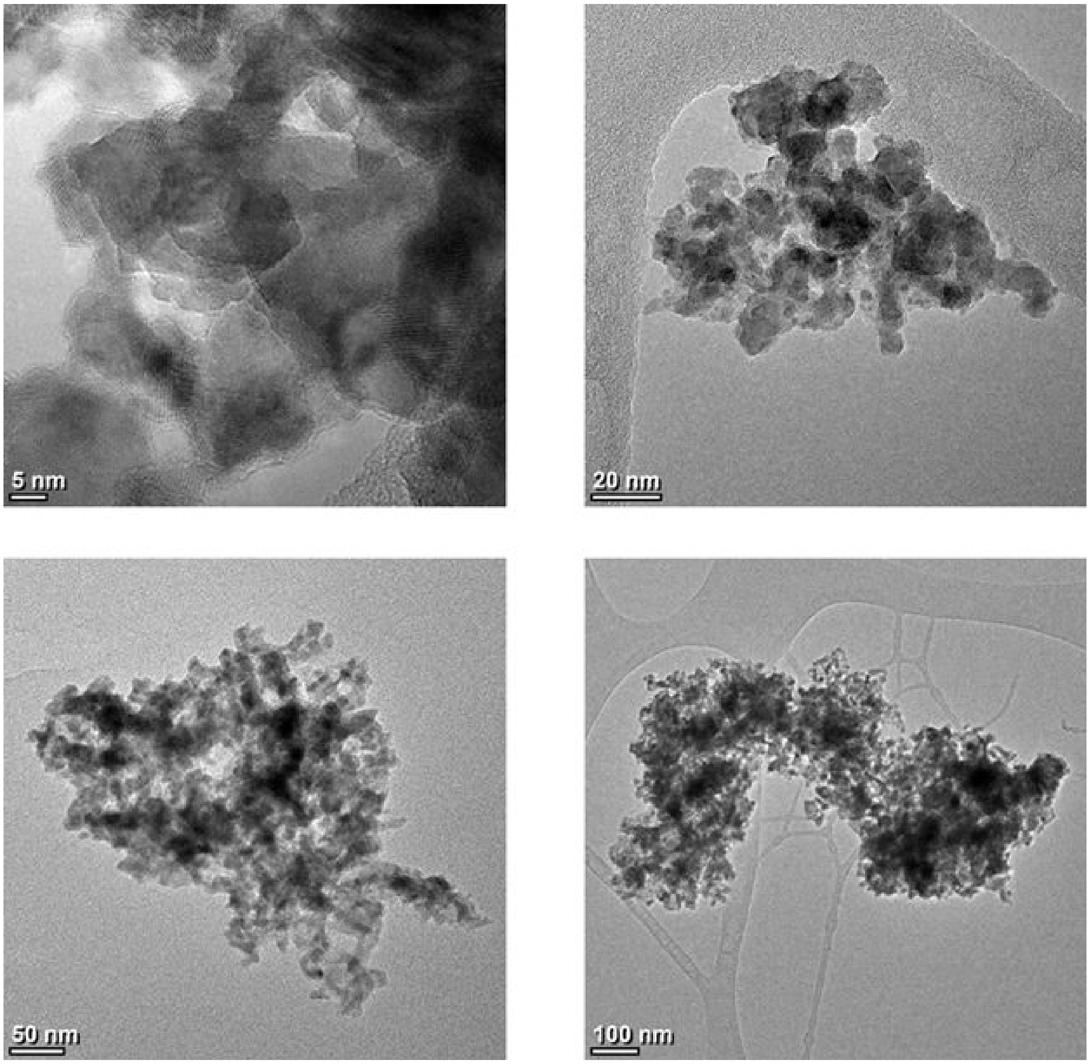

Figure S5. TEM images of Ni_3Se_4-Ni_3N/NF at different resolutions

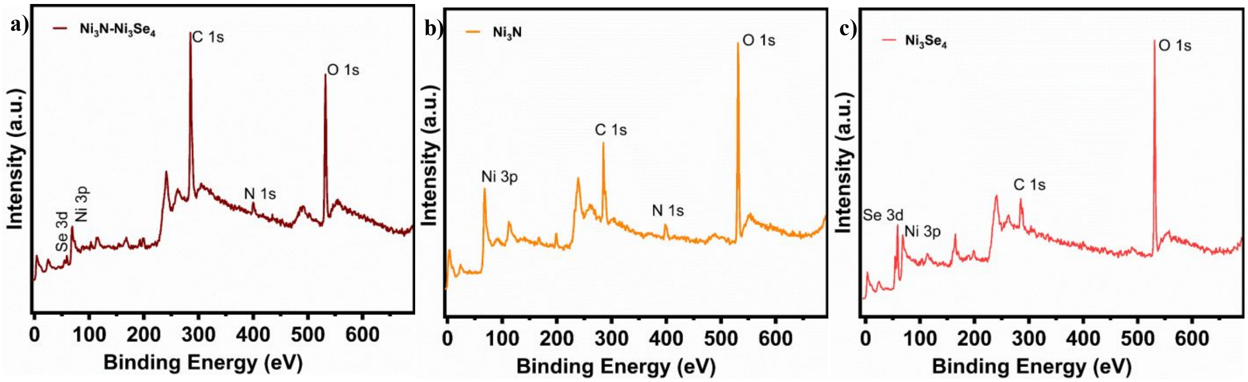

Figure S6. XPS survey spectrum for **(a)** Ni_3Se_4/NF, **(b)** Ni_3N/NF and **(c)** Ni_3Se_4-Ni_3N/NF catalysts

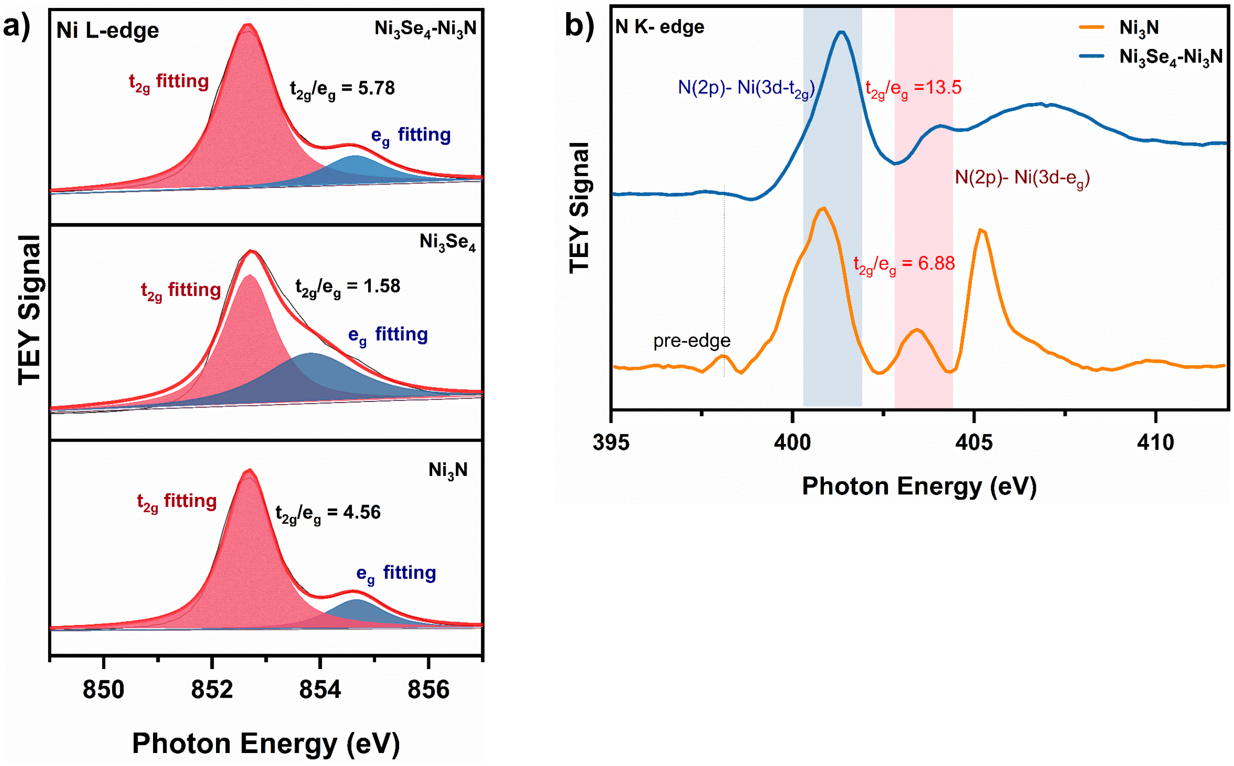

Figure S7. (a) the deconvoluted Ni L3-edge XAS spectra and (b) N K-edge XAS spectra of the catalysts

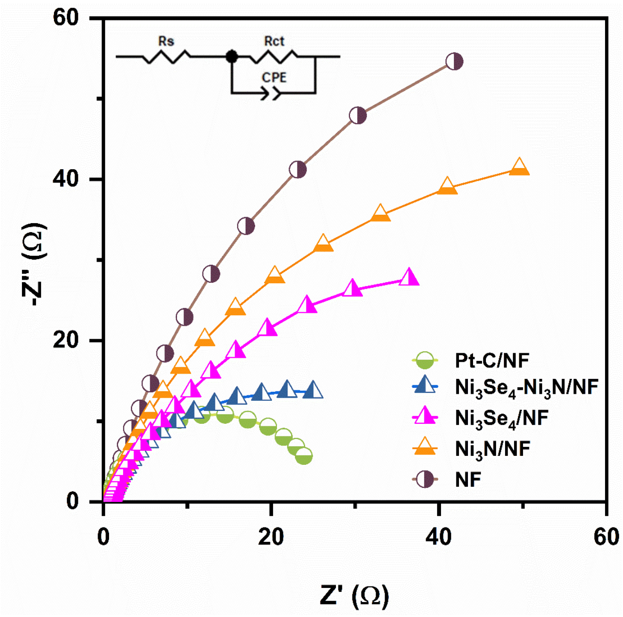

Figure S8. Nyquist plots of Ni_3N/NF, Ni_3Se_4/NF, and Ni_3Se_4-Ni_3N/NF catalysts at 50 mV overpotential.

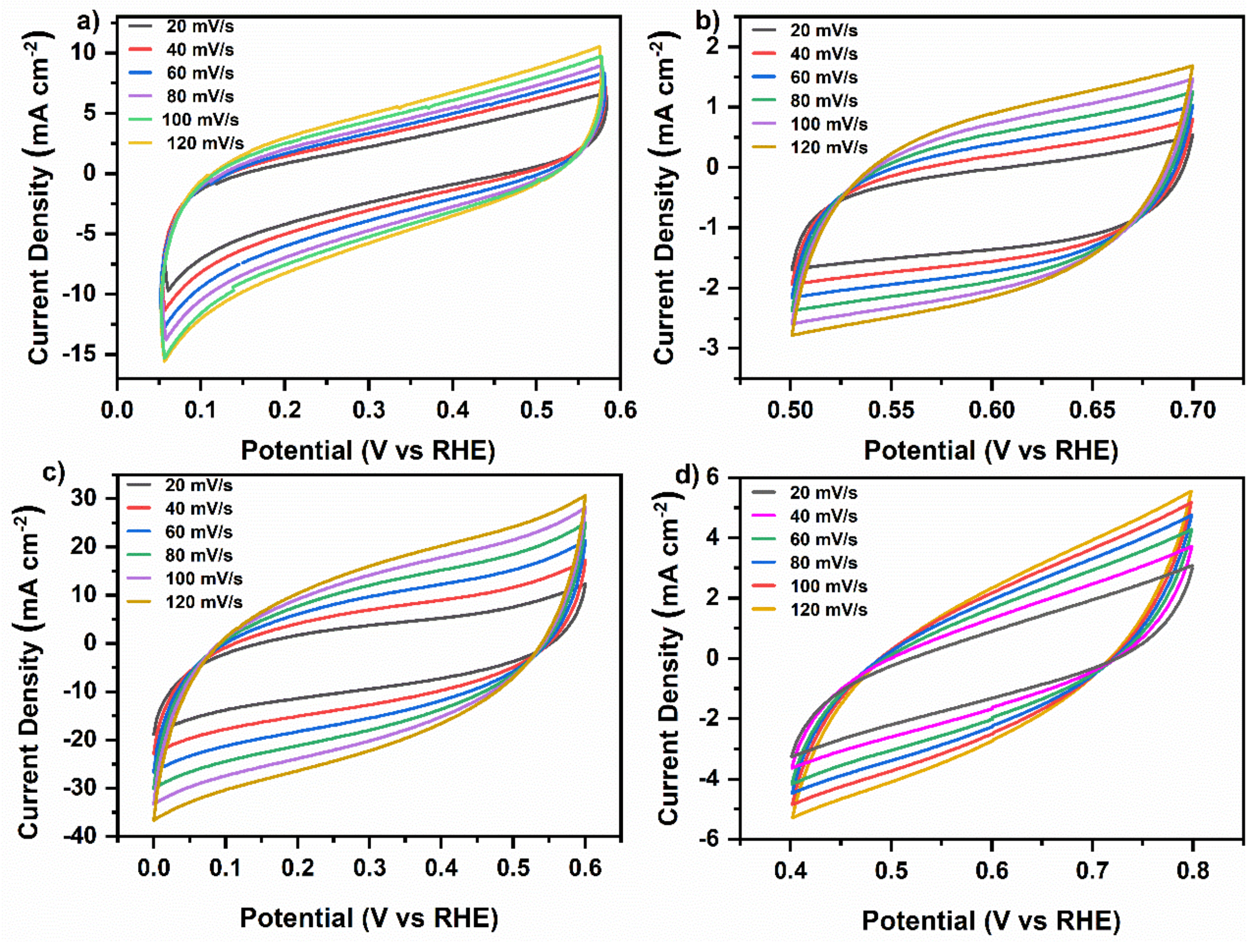

Figure S9. Cyclic voltammetry (CV) curves for Cdl of samples (a) Ni_3N/NF, (b) Ni_3Se_4/NF, (c) Ni_3Se_4-Ni_3N/NF and (d) 20 wt.% Pt-C/NF obtained at different scan rates.

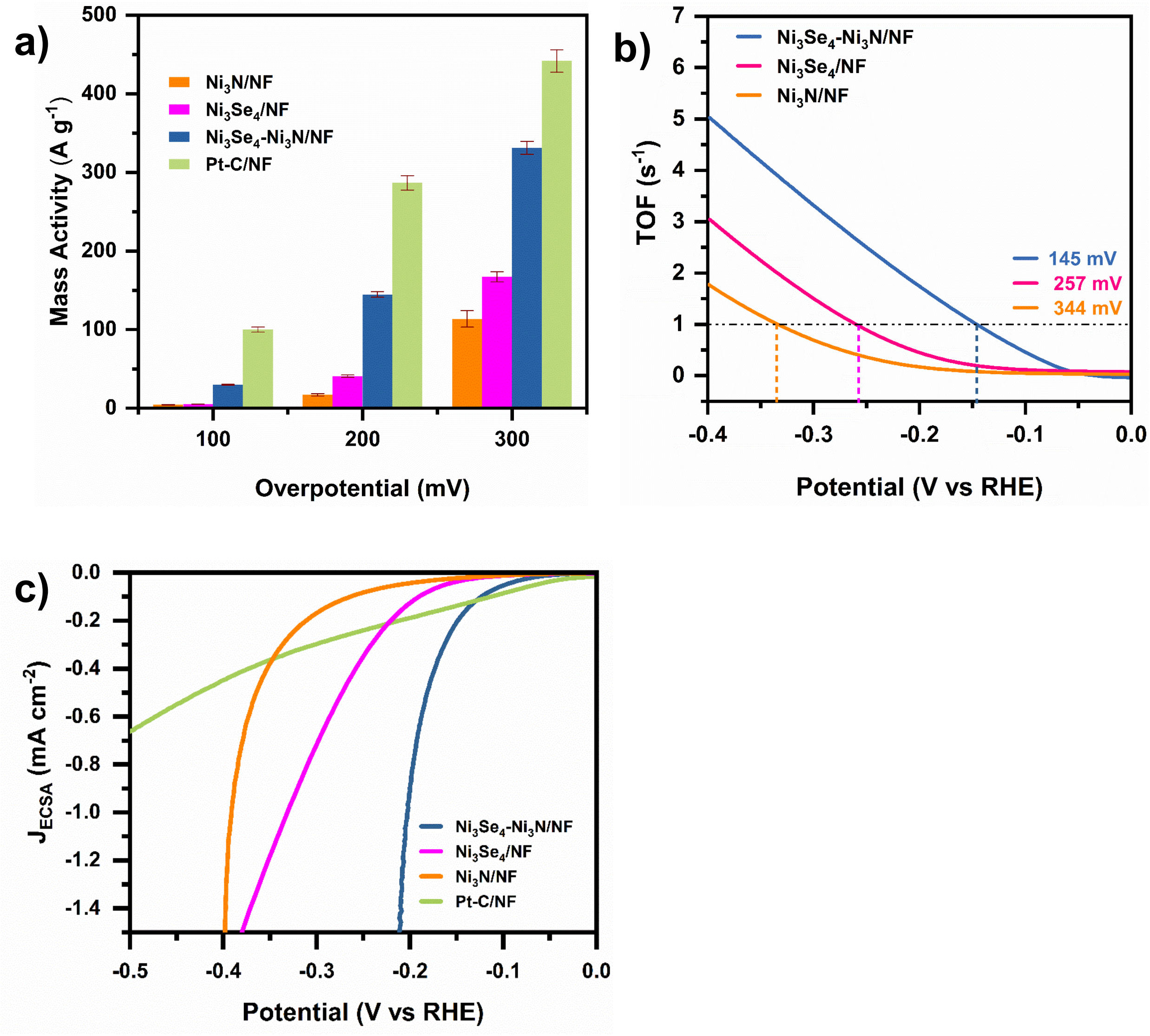

Figure S10. (a) mass activities, (b) Turn over frequency (TOF) values and (c) ECSA normalized LSV curves of the catalysts

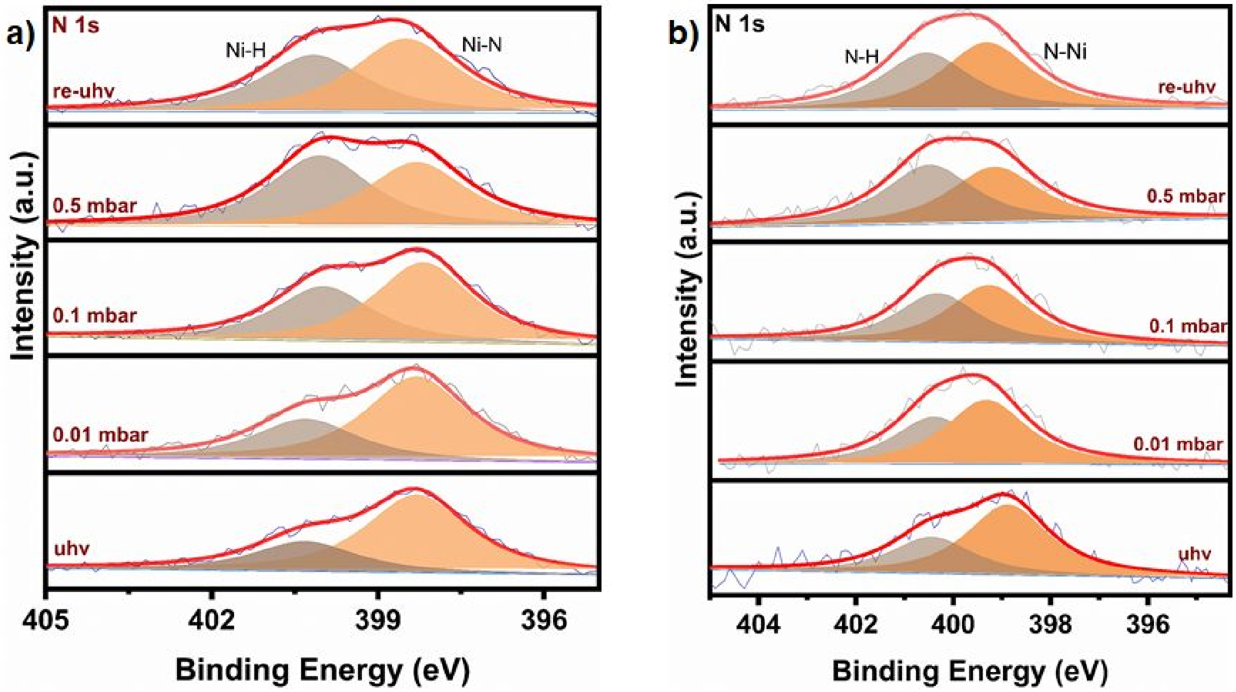

Figure S11. Water adsorption characterization using Near Ambient Pressure X-ray Photoelectron Spectroscopy: The deconvoluted HR-XPS N 1s spectra of (a) Ni_3N/NF, (b) Ni_3Se_4-Ni_3N/NF at different water pressures.

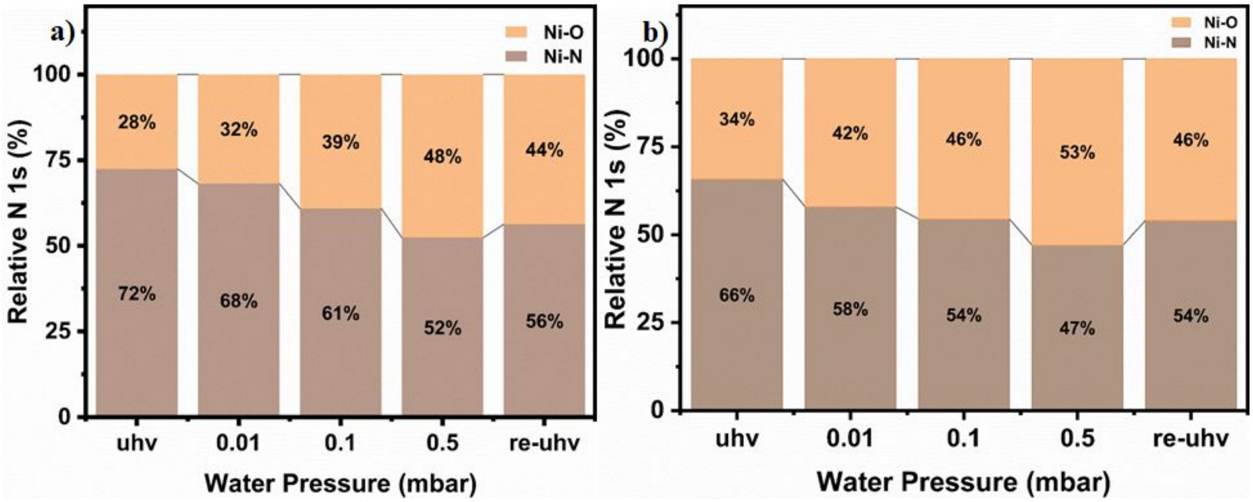

Figure S12. Water adsorption characterizations. The relative N 1s diagram of (a) Ni_3N/NF and (b) Ni_3Se_4-Ni_3N/NF electrocatalysts at different water pressure

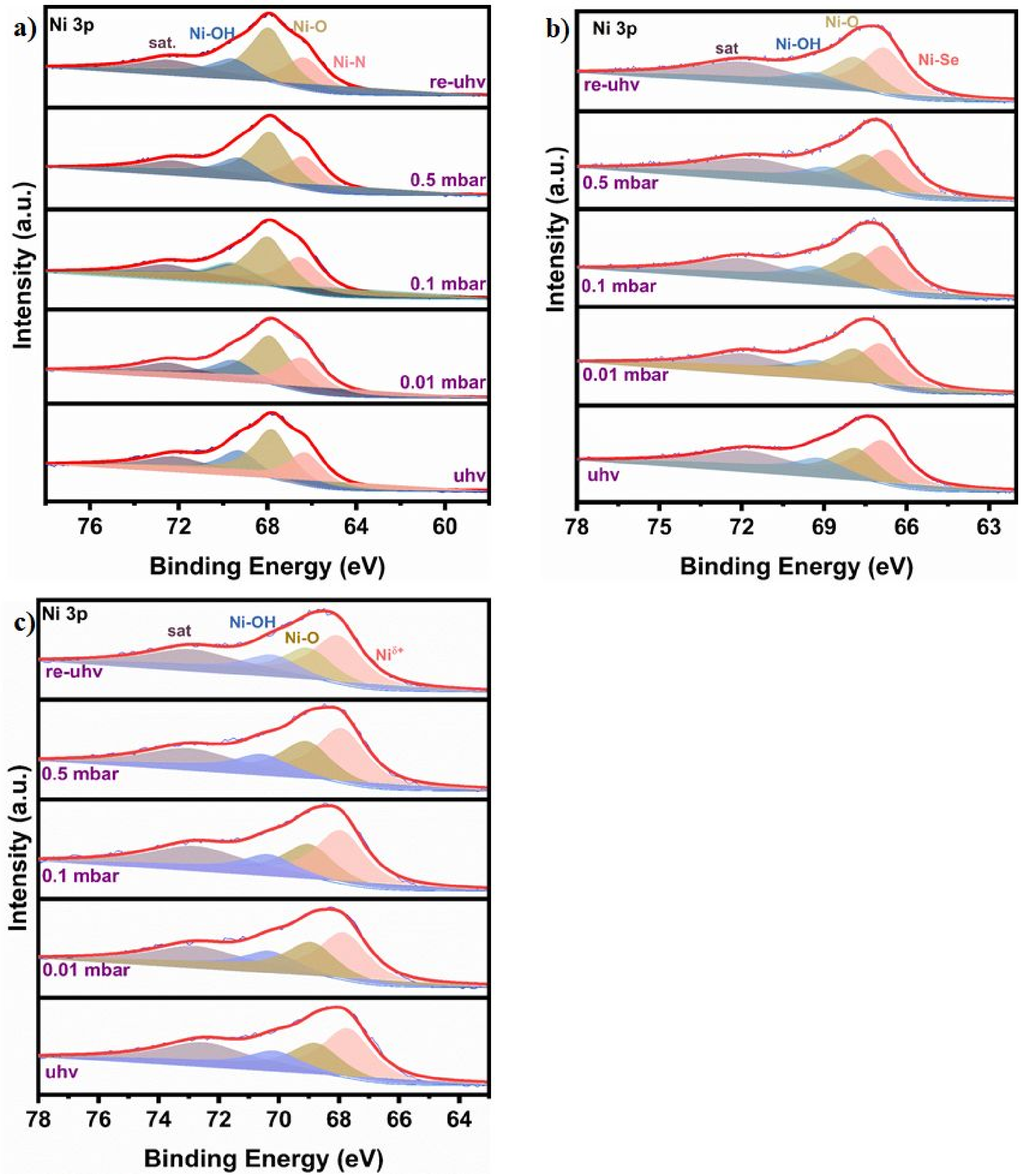

Figure S13. Water adsorption characterizations. The deconvoluted HR-XPS Ni 3p spectra of (a) Ni_3N/NF, (b) Ni_3Se_4/NF and (c) Ni_3Se_4-Ni_3N/NF electrocatalysts at different water pressure

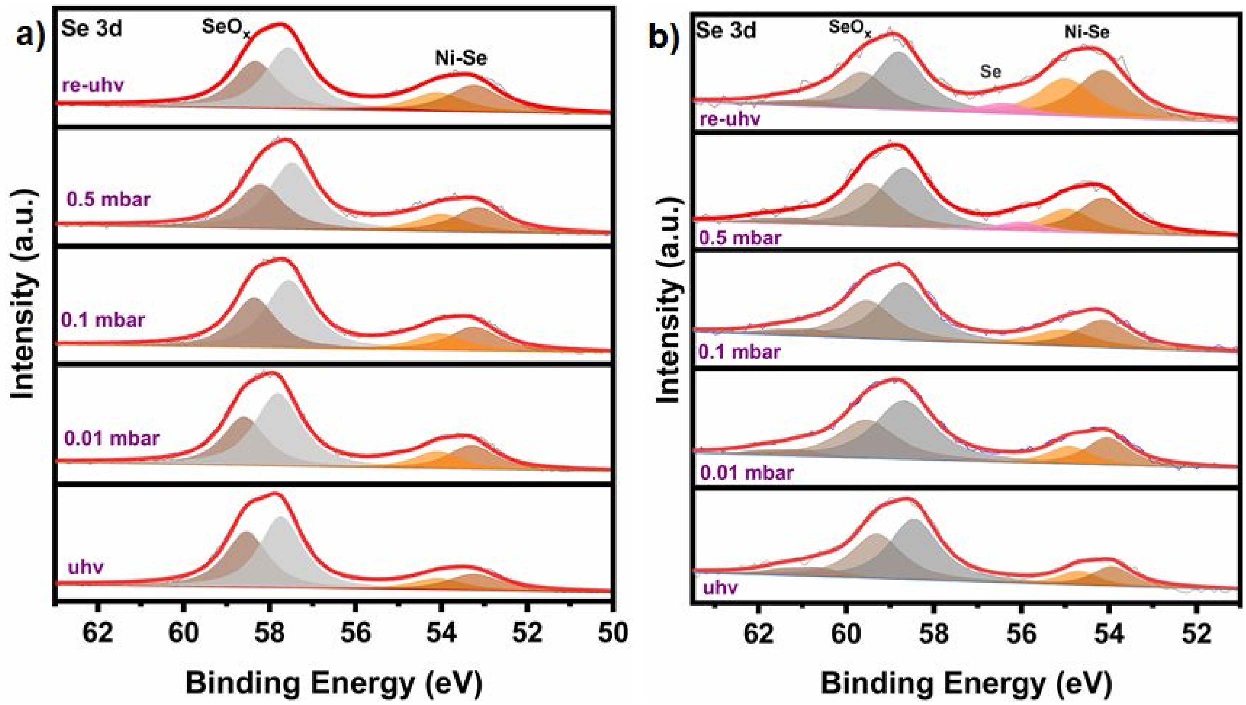

Figure S14. Water adsorption characterizations. The deconvoluted HR-XPS Se 3d spectra of (a) Ni_3Se_4/NF (b) Ni_3Se_4-Ni_3N/NF electrocatalysts at different water pressures

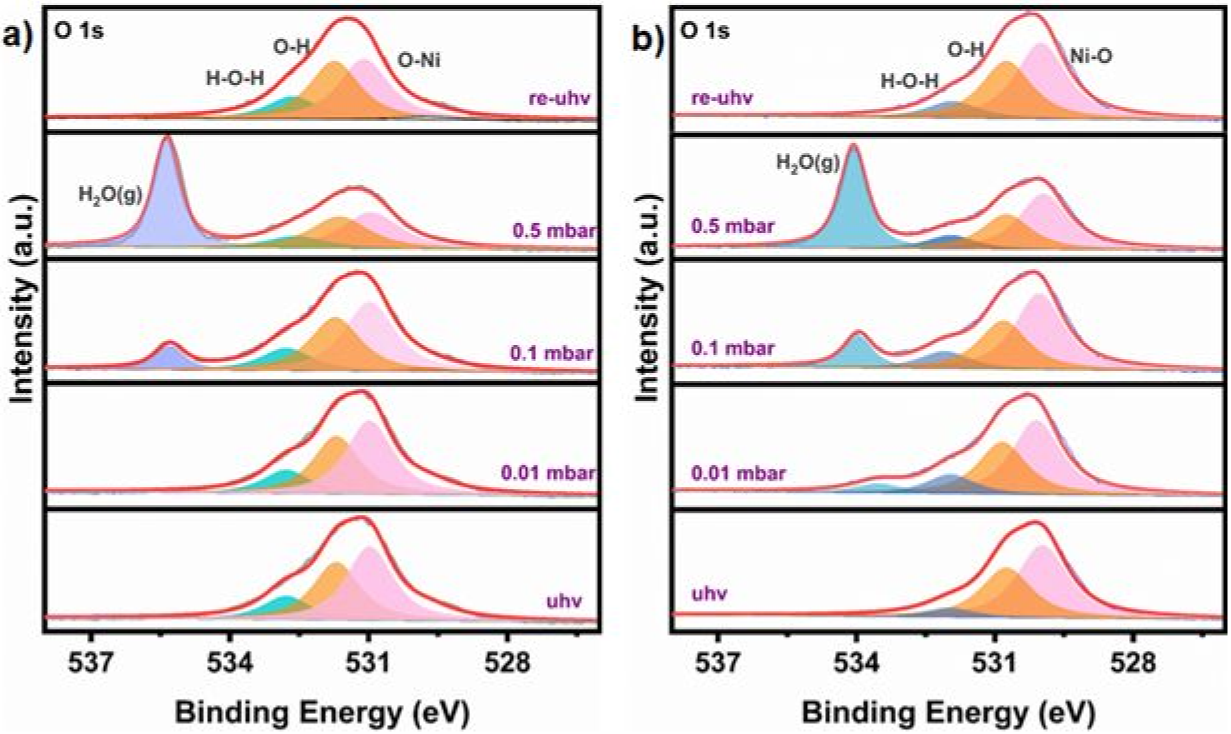

Figure S15. Water adsorption characterization using Near Ambient Pressure X-ray Photoelectron Spectroscopy: The deconvoluted HR-XPS O 1s spectra of (a) Ni_3N/NF, (b) Ni_3Se_4/NF at different water pressure.

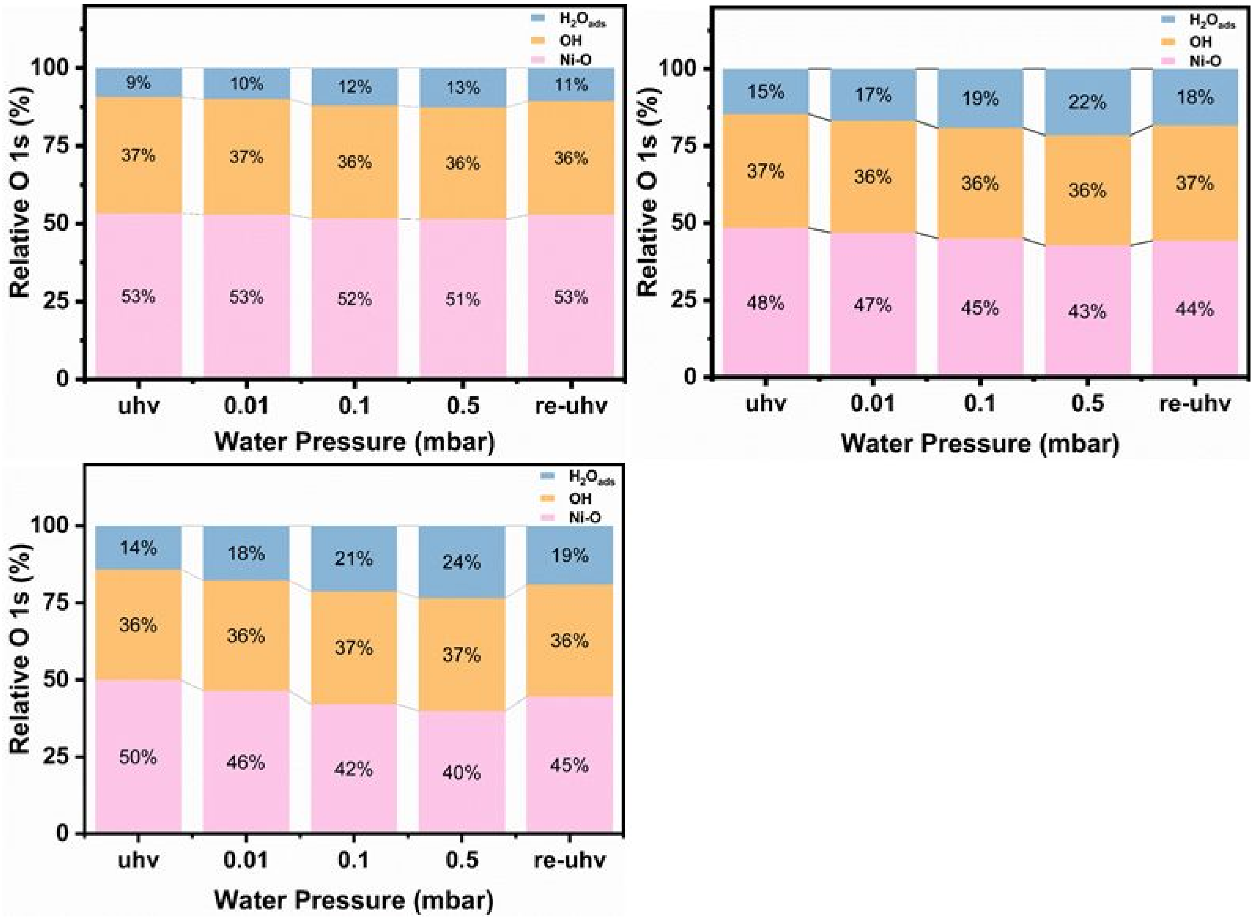

Figure S16. The relative O 1s diagram of (a) Ni_3Se_4/NF, (b) Ni_3N/NF and (c) Ni_3Se_4-Ni_3N/NF electrocatalysts at different water pressures.

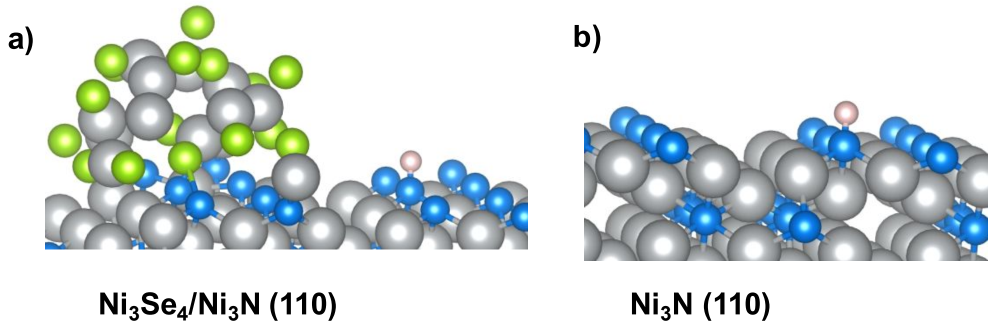

Figure S17. Atomic sites and optimized models (a) Ni_3Se_4-Ni_3N (110) and (b) Ni_3N (110) used for H* adsorption free energy determination. The gray, light green and blue balls represent Ni, Se and N atoms respectively, the small purple ball is H atom.

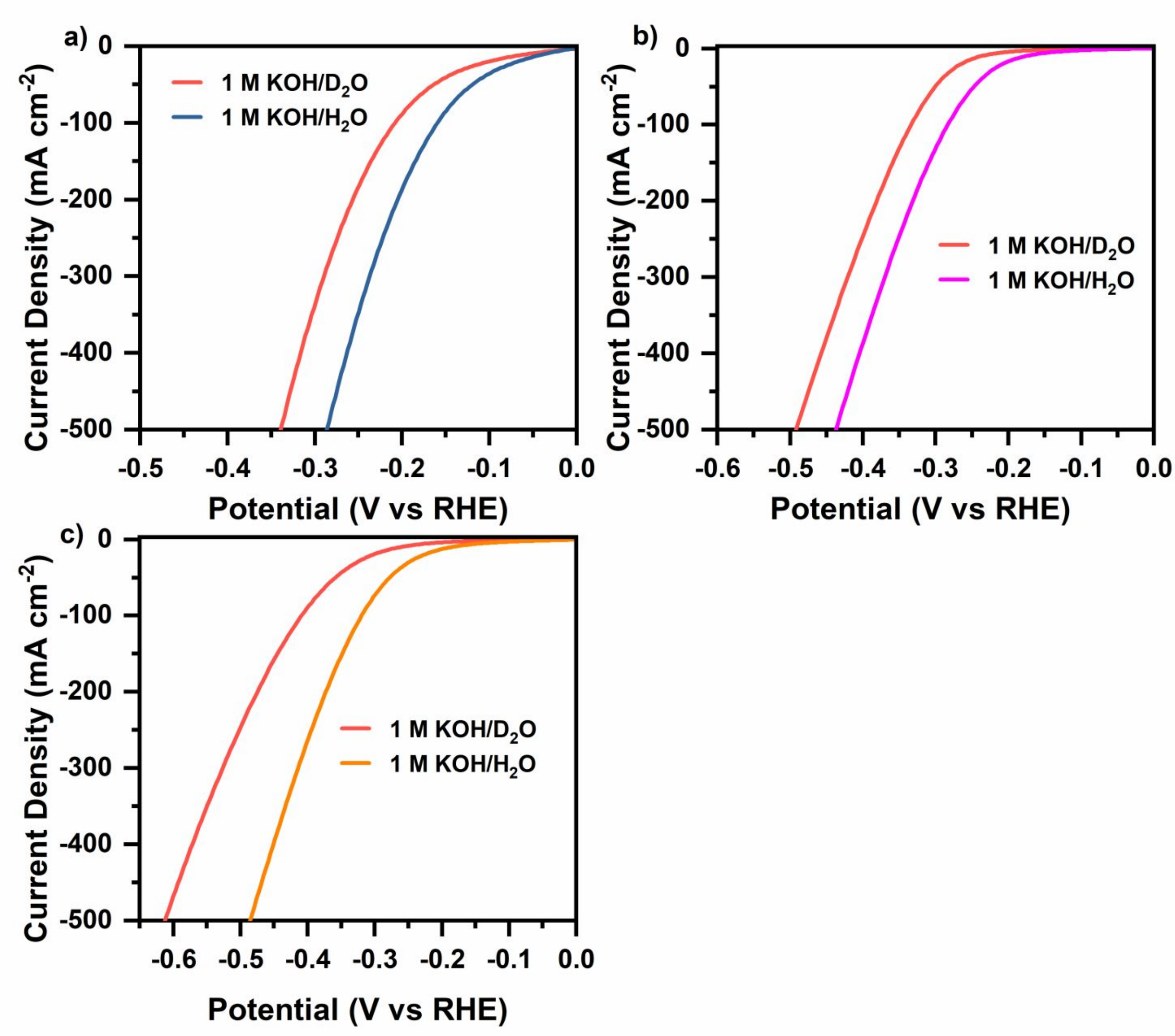

Figure S18. Polarization curves of (a) Ni_3Se_4-Ni_3N/NF, (b) Ni_3Se_4/NF and (c) Ni_3N/NF catalysts in aqueous 1 M KOH and 1 M KOH in D_2O electrolytes.

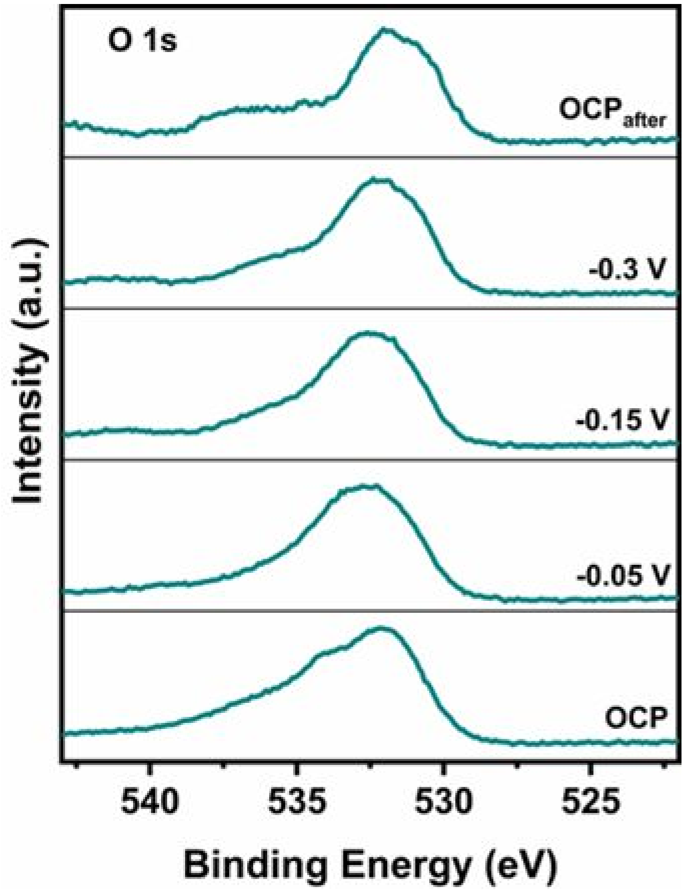

Figure S19. In-situ XPS characterization. HR-XPS O 1s spectra of Ni_3Se_4-Ni_3N/NF dual-site catalyst acquired under different applied potential in 1 M KOH electrolyte.

Supplementary Table S1. The HER performance of each catalyst

Samples	Mass loading ($mg\ cm^{-2}$)	ECSA (cm^2)	η_{10} (mV)	Tafel Slope ($mV\ dec^{-1}$)	Rs (Ω)	Rct (Ω)
**Pt-C/NF**	0.25	505	41	40.11	0.3	27.62
**Ni_3Se_4-Ni_3N/NF**	1.57	1177.5	60	51.10	0.24	7.82
**Ni_3Se_4/NF**	1.34	467.5	145	64.61	0.25	10.49
**Ni_3N/NF**	1.17	770	195	65.86	0.22	17.97
**NF**	-	-	290	94.95	0.3	-

Supplementary Table S2. The KIE of the electrocatalysts calculated at different overpotentials

	**Ni_3S_4-Ni_3N/NF**			**Ni_3Se_4/NF**			**Ni_3N/NF**		
**J_{H2O}**	**η (mV)**	**J_{D2O}**	**KIE**	**η (mV)**	**J_{D2O}**	**KIE**	**η (mV)**	**J_{D2O}**	**KIE**
**50**	120	26.37	1.896	220	14.30	3.495	270	14.27	3.496
**100**	160	46.91	2.132	258	34.68	2.890	319	25.49	3.922
**150**	185	69.90	2.152	283	63.51	2.362	347	40.71	3.685
**200**	204	97.68	2.047	304	95.51	2.094	372	61.92	3.231

Supplementary Table S3. Density functional theory (DFT) simulated free energies of adsorption of intermediates on varies sites on Ni_3N and Ni_3Se_4-Ni_3N heterointerfaces systems in alkaline electrolyte.

Systems	ΔG_H (eV) (site)	ΔG_{OH} (eV) (site)
Ni_3N (110)	-0.345 (Ni-b)	-0.129 (Ni-b)
	-0.523 (Ni-b-sub)	0.211 (Ni-b-sub)
	-0.534 (Ni-h1)	-0.044 (Ni-h1)
	-0.276 (Ni-h2)	0.432 (Ni-h2)
	0.076 (Ni-top)	0.437 (Ni-top)
	-0.531 (Ni-top-sub)	0.130 (Ni-top-sub)
	-0.705 (N site)	
Ni_3Se_4/Ni_3N (110)	0.648 (1, iNi-NiSe)	1.764 (iNi-NiSe)
	-0.465 (2, iNiNSe)	0.174 (iNiNSe)
	-0.204 (3, iNiSe)	0.589 (iNiSe)
	0.493 (4, iSe-NiSe)	1.779 (iSe-NiSe)
	0.543 (5, Ni-NiSe)	1.326 (Ni-NiSe)
	-0.027 (6, Se-NiSe)	1.160 (Se-NiSe)
	-0.707 (N site)	

Supplementary Table S4. Comparisons of the catalytic performances of Ni_3Se_4-Ni_3N in this work with other reported Se-based electrocatalysts in 1 M KOH.

Electrocatalyst	Overpotential (mV@ 10 mA cm^{-2})	Tafel slope (mV dec^{-1})	Reference
**Ni_3Se_4-Ni_3N/NF**	60	51.1	This work
**Mo-$Co_{0.85}Se_{VSe}$/NC**	151	51	[6]
**Pt/np-$Co_{0.85}$Se**	55	35	[7]
**Mo−Fe(1/1)−Se−CP**	86.9	57.7	[8]
**Ni_2P-$NiSe_2$**	66	72.5	[9]
**Ni_3Se_4/Ni**	106	74	[10]
**$MoSe_2$/NiSe**	210	56	[11]
**S-$Co_{0.85}$Se**	108	59	[12]
**1T-MoSe2/ NiSe NS/NW**	120	86	[13]
**Se-$NiSe_2$**	117	32	[14]
**$Co_{0.75}Ni_{0.25}$Se/NF**	106	58	[15]
**$MoSe_2$@WSe_2**	231	87	[16]
**$NiSe_2$/$MoSe_2$@CC**	175	78	[17]
**$Ni_{0.85}$Se/$MoSe_2$**	124	63	[18]
**$Co_{0.1}Ni_{0.75}$Se/rGO**	103	42	[19]
**CuNi/NiSe**	41	42	[20]
**CuS@MoSe2**	72	77.6	[21]
**Co0.85Se/MoSe2**	70	70	[22]

References

1. Zhang, R.; Wang, X.; Yu, S.; Wen, T.; Zhu, X.; Yang, F.; Sun, X.; Wang, X.; Hu, W., Ternary $NiCo_2P_x$ Nanowires as pH-Universal Electrocatalysts for Highly Efficient Hydrogen Evolution Reaction. *Adv Mater* **2017,** *29* (9), 1605502.
2. Jin, M.; Zhang, X.; Shi, R.; Lian, Q.; Niu, S.; Peng, O.; Wang, Q.; Cheng, C., Hierarchical CoP@Ni_2P catalysts for pH-universal hydrogen evolution at high current density. *Appl. Catal. B* **2021,** *296*, 120350.
3. Moges, E. A.; Chang, C.-Y.; Huang, W.-H.; Lakshmanan, K.; Awoke, Y. A.; Pao, C.-W.; Tsai, M.-C.; Su, W.-N.; Hwang, B. J., Sustainable Synthesis of Dual Single-Atom Catalyst of Pd-N_4/Cu-N_4 for Partial Oxidation of Ethylene Glycol. *Adv. Funct. Mater.* **2022,** *32* (46), 2206887.
4. Kresse, G.; Hafner, J., Ab initio molecular dynamics for liquid metals. *Physical review B* **1993,** *47* (1), 558.
5. Kresse, G.; Joubert, D., From ultrasoft pseudopotentials to the projector augmented-wave method. *Physical review b* **1999,** *59* (3), 1758.
6. Dai, Q.; Wang, L.; Wang, K.; Sang, X.; Li, Z.; Yang, B.; Chen, J.; Lei, L.; Dai, L.; Hou, Y., Accelerated water dissociation kinetics by electron-enriched cobalt sites for efficient alkaline hydrogen evolution. *Advanced Functional Materials* **2022,** *32* (12), 2109556.
7. Jiang, K.; Liu, B.; Luo, M.; Ning, S.; Peng, M.; Zhao, Y.; Lu, Y.-R.; Chan, T.-S.; de Groot, F. M.; Tan, Y., Single platinum atoms embedded in nanoporous cobalt selenide as electrocatalyst for accelerating hydrogen evolution reaction. *Nature communications* **2019,** *10* (1), 1743.
8. Chen, Y.; Zhang, J.; Guo, P.; Liu, H.; Wang, Z.; Liu, M.; Zhang, T.; Wang, S.; Zhou, Y.; Lu, X., Coupled heterostructure of Mo–Fe selenide nanosheets supported on carbon paper as an

integrated electrocatalyst for efficient hydrogen evolution. *ACS applied materials & interfaces* **2018,** *10* (33), 27787-27794.

9. Liu, C.; Gong, T.; Zhang, J.; Zheng, X.; Mao, J.; Liu, H.; Li, Y.; Hao, Q., Engineering Ni2P-NiSe2 heterostructure interface for highly efficient alkaline hydrogen evolution. *Applied Catalysis B: Environmental* **2020,** *262*, 118245.
10. Guo, K.; Wang, Y.; Huang, J.; Li, H.; Peng, Y.; Xu, C., Symbiotic Ni3Se4/Ni heterostructure induced by unstable NiSe2 for enhanced hydrogen generation. *Chemical Engineering Journal* **2023,** *454*, 140488.
11. Zhou, X.; Liu, Y.; Ju, H.; Pan, B.; Zhu, J.; Ding, T.; Wang, C.; Yang, Q., Design and epitaxial growth of MoSe2–NiSe vertical heteronanostructures with electronic modulation for enhanced hydrogen evolution reaction. *Chemistry of Materials* **2016,** *28* (6), 1838-1846.
12. Shen, S.; Lin, Z.; Song, K.; Wang, Z.; Huang, L.; Yan, L.; Meng, F.; Zhang, Q.; Gu, L.; Zhong, W., Reversed active sites boost the intrinsic activity of graphene-like cobalt selenide for hydrogen evolution. *Angewandte Chemie* **2021,** *133* (22), 12468-12473.
13. Zhang, X.; Zhang, Y. Y.; Zhang, Y.; Jiang, W. J.; Zhang, Q. H.; Yang, Y. G.; Gu, L.; Hu, J. S.; Wan, L. J., Phase-controlled synthesis of 1T-MoSe2/NiSe heterostructure nanowire arrays via electronic injection for synergistically enhanced hydrogen evolution. *Small Methods* **2019,** *3* (2), 1800317.
14. Wang, F.; Li, Y.; Shifa, T. A.; Liu, K.; Wang, F.; Wang, Z.; Xu, P.; Wang, Q.; He, J., Selenium-enriched nickel selenide nanosheets as a robust electrocatalyst for hydrogen generation. *Angewandte Chemie International Edition* **2016,** *55* (24), 6919-6924.

15. Liu, S.; Jiang, Y.; Yang, M.; Zhang, M.; Guo, Q.; Shen, W.; He, R.; Li, M., Highly conductive and metallic cobalt–nickel selenide nanorods supported on Ni foam as an efficient electrocatalyst for alkaline water splitting. *Nanoscale* **2019,** *11* (16), 7959-7966.

16. Rai, R. K.; Sarkar, B.; Ram, R.; Nanda, K. K.; Ravishankar, N., Designed synthesis of a hierarchical MoSe 2@ WSe 2 hybrid nanostructure as a bifunctional electrocatalyst for total water-splitting. *Sustainable Energy & Fuels* **2022,** *6* (7), 1708-1718.

17. Zhu, M.; Yan, Q.; Xue, Y.; Yan, Y.; Zhu, K.; Ye, K.; Yan, J.; Cao, D.; Xie, H.; Wang, G., Free-standing P-doped NiSe2/MoSe2 catalyst for efficient hydrogen evolution in acidic and alkaline media. *ACS Sustainable Chemistry & Engineering* **2021,** *10* (1), 279-287.

18. Li, R.; Xie, S.; Zeng, Y.; Zhao, Q.; Mao, M.; Liu, Z.; Chu, P. K.; Peng, X., Synergistic dual-regulating the electronic structure of NiMo selenides composite for highly efficient hydrogen evolution reaction. *Fuel* **2024,** *358*, 130203.

19. Zhao, W.; Wang, S.; Feng, C.; Wu, H.; Zhang, L.; Zhang, J., Novel Cobalt-Doped Ni0. 85Se Chalcogenides (Co x Ni0. 85–x Se) as High Active and Stable Electrocatalysts for Hydrogen Evolution Reaction in Electrolysis Water Splitting. *ACS Applied Materials & Interfaces* **2018,** *10* (47), 40491-40499.

20. Cao, D.; Shao, J.; Cui, Y.; Zhang, L.; Cheng, D., Interfacial engineering of copper–nickel selenide nanodendrites for enhanced overall water splitting in alkali condition. *Small* **2023,** *19* (33), 2301613.

21. Gu, M.; Jiang, L.; Zhao, S.; Wang, H.; Lin, M.; Deng, X.; Huang, X.; Gao, A.; Liu, X.; Sun, P., Deciphering the space charge effect of the p–n junction between copper sulfides and molybdenum selenides for efficient water electrolysis in a wide pH range. *ACS nano* **2022,** *16* (9), 15425-15439.

22. Banerjee, K.; Roy, A.; Ghosh, S.; Inta, H. R.; Mondal, A.; Ghosh, S.; Mitra, A.; Mahato, A. K.; Mahalingam, V., Revitalizing Alkaline Hydrogen Evolution Reaction Performance of Co0. 85Se/MoSe2 by Microstructural Engineering with Nonprecious Cobalt Oxalate. *ACS Applied Energy Materials* **2024,** *7* (18), 7745-7758.
